# Vestibular Deficits in Deafness: Clinical Presentation, Animal Modeling, and Treatment Solutions

**DOI:** 10.3389/fneur.2022.816534

**Published:** 2022-04-04

**Authors:** Audrey Maudoux, Sandrine Vitry, Aziz El-Amraoui

**Affiliations:** ^1^Unit Progressive Sensory Disorders, Pathophysiology and Therapy, Institut Pasteur, Institut de l'Audition, Université de Paris, INSERM-UMRS1120, Paris, France; ^2^Center for Balance Evaluation in Children (EFEE), Otolaryngology Department, Assistance Publique des Hôpitaux de Paris, Robert-Debré University Hospital, Paris, France

**Keywords:** balance disorders, vestibulopathy, rare diseases, animal models, pathophysiology and gene therapy, deafness

## Abstract

The inner ear is responsible for both hearing and balance. These functions are dependent on the correct functioning of mechanosensitive hair cells, which convert sound- and motion-induced stimuli into electrical signals conveyed to the brain. During evolution of the inner ear, the major changes occurred in the hearing organ, whereas the structure of the vestibular organs remained constant in all vertebrates over the same period. Vestibular deficits are highly prevalent in humans, due to multiple intersecting causes: genetics, environmental factors, ototoxic drugs, infections and aging. Studies of deafness genes associated with balance deficits and their corresponding animal models have shed light on the development and function of these two sensory systems. Bilateral vestibular deficits often impair individual postural control, gaze stabilization, locomotion and spatial orientation. The resulting dizziness, vertigo, and/or falls (frequent in elderly populations) greatly affect patient quality of life. In the absence of treatment, prosthetic devices, such as vestibular implants, providing information about the direction, amplitude and velocity of body movements, are being developed and have given promising results in animal models and humans. Novel methods and techniques have led to major progress in gene therapies targeting the inner ear (gene supplementation and gene editing), 3D inner ear organoids and reprograming protocols for generating hair cell-like cells. These rapid advances in multiscale approaches covering basic research, clinical diagnostics and therapies are fostering interdisciplinary research to develop personalized treatments for vestibular disorders.

## Introduction

**Figure 1:**
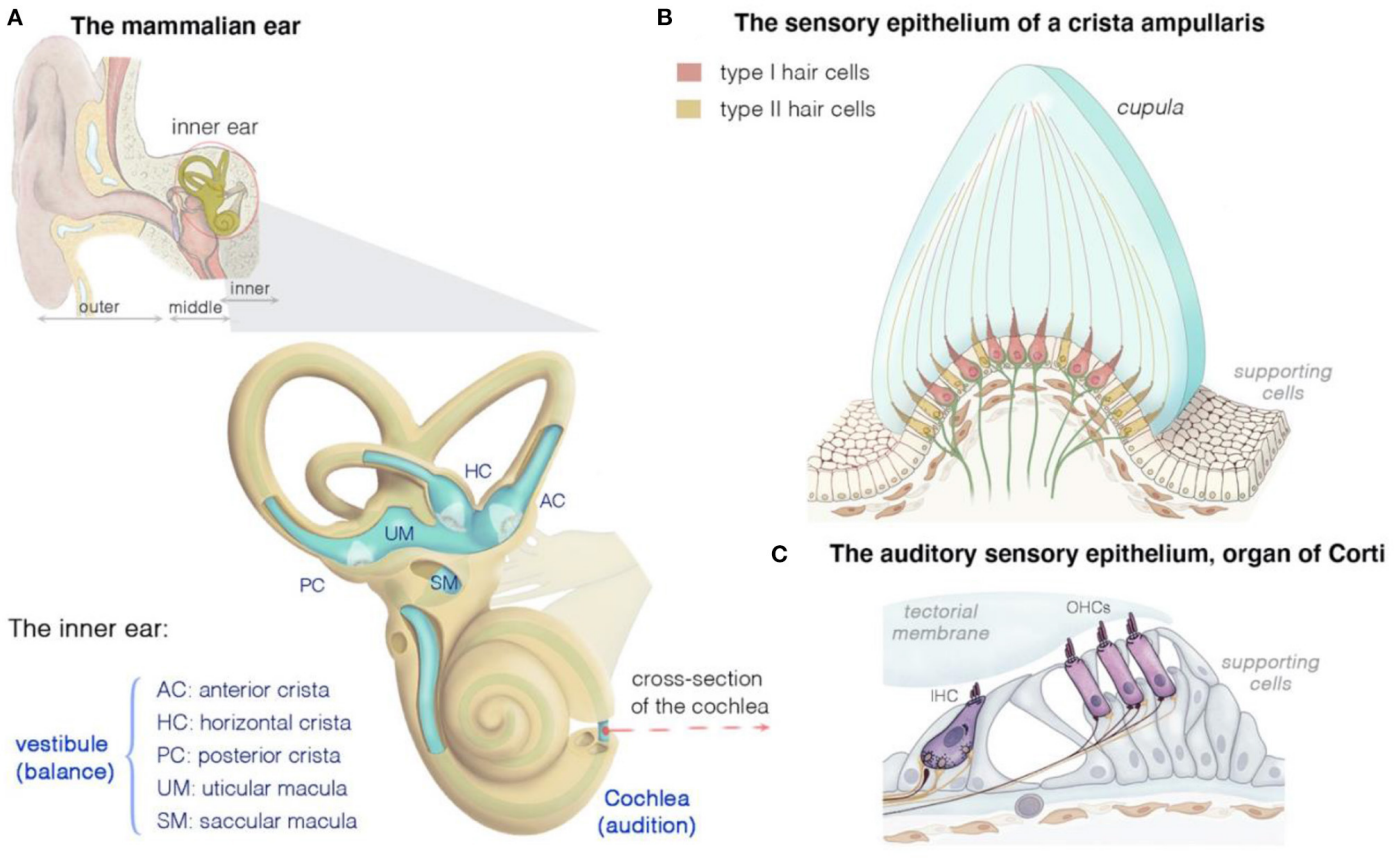
Structure and function of the mammalian inner ear, and of the organization of the vestibular and auditory sensory epithelia. **(A)** The inner ear houses both the hearing and the balance organs, the cochlea and the vestibule, respectively. The membranous labyrinth of the inner ear consists of three semicircular canals, each ending with an ampulla that form the anterior (AC), horizontal (HC), or posterior (PC) crista, two otolith organs (saccule and utricle), and the cochlea. The calyx- and bouton-like afferent innervation of hair cells is presented in green. **(B)** Cross section of a crista ampullaris, highlighting the organization of the type I and type II hair cells. All the crista hair cells have hair bundles displaying the same polarity orientation, extending into a gelatinous membrane called the cupula. Details of the macular sensory regions' organization are shown in Figure 2. **(C)** Cross section of the organ of Corti, showing the inner (IHC) and outer (OHCs) hair cells, associated afferent (green) and efferent (dark purple red) neurons and supporting cells.

Our sensory organs mediate and control our responses to the external and internal environment, continuously adapting to its various changes. The human inner ear, one of the major organs signaling to the central nervous system, consists of spatially interconnected fluid-filled ducts and chambers housing six different neurosensory suborgans essential for balance and hearing (Figure 1). The resulting labyrinth is one of the most morphologically elaborate tissues in vertebrates. Its intricate and fascinating organization and capacity to integrate sensory perceptions from multiple sources have, over the centuries, triggered considerable interest leading to research across many disciplines, including physics, developmental biology, neuroscience, behavioral cognition, genetics, organ evolution, molecular phylogeny, engineering, and clinical and translational medicine.

Hearing and balance display tight anatomical and functional links. Both organs originated from a single otic vesicle that develops by the invagination of surface ectoderm adjacent to the developing hindbrain (1–5). However, changes in the needs of species in fluctuating environments have led to species-specific tissue and cellular adaptations. The auditory system has always been a subject of great interest and the focus of extensive research, due to its key role in the evolution of language and sound-based communication (1, 6). By comparison, at least until recently, the vestibular system has received much less attention. The inner ear, which first emerged about 500 million years ago, was initially composed exclusively of a gravitational organ that evolved to sense gravity, and then motion and balance (7). In humans, defects of hearing and/or balance, of peripheral and/or central origin, may manifest at any age, with varying degrees of severity. Vestibular dysfunction leads to one of the most common complaints in medicine, dizziness and/or vertigo, which affects 15–35% of the general population (8–11). In Europe, vestibular disorders of peripheral origin account for 3% of all medical consultations for patients over the age of 50 years and about 1% of the emergency cases seen at hospitals (12, 13). National questionnaires have estimated that the prevalence of dizziness/vertigo in children (3 years of age to adolescence) ranges from 5.3 to 8% (14, 15). However, these figures are probably underestimated, as young children tolerate dizziness better than adolescents and adults. In clinics specializing in the treatment of pediatric vestibular disorders, the prevalence of vestibular dysfunction in children referred for dizziness/vertigo ranges from 20 to 36.5% (16, 17). The prevalence of balance dysfunction increases with age; about one third of adults over the age of 65 years suffers from dizziness, resulting in frequent falls (18).

Over the last 20 years, an increasing awareness of the actual importance of the vestibular system has fueled substantial work that has shed light on the multifaceted contributions of this system. The inputs of the vestibular sensory organs extend far beyond the brainstem, reaching many cortical centers in the brain. Indeed, vestibular system activity supports numerous functions, from gaze stabilization and postural control to high-level cortical functions involving spatial cognition, including self-body perception, verticality perception, orientation, navigation, and spatial memory (19–22). The vestibular organs also influence postural blood pressure regulation, bone density and muscle composition via specific vestibulo-sympathetic pathways in the brainstem and mesencephalon and have been shown to act as a powerful synchronizer of circadian rhythms and play a substantial role in sleep-wake cycles (7). Untreated losses of vestibular function have a major effect on patient quality of life and a major socioeconomic impact on healthcare systems worldwide.

Despite the high level of medical need, the management of patients with vertigo and dizziness still suffers from a lack of diagnostic tools for accurately monitoring balance and its effects on the development and behavior of the individual. In children, the timely detection of vestibular dysfunction is crucial, to provide parents with appropriate counseling and for the initiation of appropriate vestibular rehabilitation. Biomarkers for the more accurate diagnosis of vestibular disorders and the selection of targeted therapies are needed but not yet available. The pathophysiological mechanisms underlying the various vestibular disorders also remain unknown and there are currently no curative solutions. Redundancy and compensatory mechanisms may operate in cases of vestibular organ dysfunction, but the magnitude and accuracy of peripheral and central multisensory and behavioral substitution processes, and of the plasticity mechanisms sustaining the restoration of activity remain unclear (23).

In this review, we address these issues with the aim of providing an integrative view of the development and function of the vestibular system, taking into account much of the knowledge accumulated to date in animal models and human individuals with and without vestibular disorders. A precise understanding of organ evolution, development, physiology, and behavior will pave the way for better diagnostic tools and the precise and accurate definition and classification of vestibular disorders. Together, these discoveries should help guide patient management and the search for effective rehabilitation and treatment strategies.

## How Does the Inner Ear Vestibular System Develop and Function?

The inner ear provides a remarkably rich context for studying cell fate and tissue organogenesis. Over the last two decades, a wealth of information has been obtained concerning how a single otic vesicle gives rise to distinct subsensory regions containing different assemblies of highly specialized hair cells, neurons, and supporting cells.

### From the Otic Vesicle to Distinct Sensory Suborgans

Gene expression data, lineage tracing, fate-mapping studies, and the characterization of animal models that display abnormal development or dysfunction in the vestibular and cochlear organs have all proved essential for unraveling the key mechanisms underlying inner ear development (2, 4, 24–26). Studies in fish, amphibian, chicken, and mouse animal models, either normal or with defects, have provided considerable insight into the signals and pathways governing the identity, proliferation, and differentiation of inner ear cells, and tissue planar polarity. Specific details about these various aspects of development are provided by many excellent recent reviews (27–29). Remarkable progress has been made in our understanding of how different morphogens and transcription factors cooperate to establish anteroposterior and dorsoventral organ patterning, mediate cell-fate commitment into sensory, non-sensory, and neurogenic components, and orchestrate correct cellular remodeling and assembly during organ morphogenesis. Various genes, including different factors (e.g., retinoid acids, cyclins), morphogens (e.g. *FGF, BMP, Notch, Wnt*), transcription factors and key molecules (e.g., *Atoh1, Gfi1, prox1, Lfng, Sox2*, and *Ngn1, NeuroD, Tbx1, Gata, Lmx, Emx, Shh, Eya1, Six1, Delta-like 1, Jagged-1 or Tlx3*/*Rnx*) (27, 28, 30–34) have been implicated in these processes. Neurotrophins (BDNF, *TrkB, TrkC*, NT3), chemoattractants (e.g., Ntn1), repellants (slits, semaphorins and ephrins) and their receptors have also been shown to play a fundamental role in early guidance decisions and the wiring of the hair-cell circuitry and associated cochlear and vestibular neuronal connections (35–38). These factors, operating alone or in specific combinations, act in a precise, well-orchestrated sequence, at specific timepoints and positions within the organ, to facilitate the sequential cell commitment, differentiation, and tissue integration of each cell type in the appropriate position. Further studies are required to improve our understanding of the interdependent sequences of the developmental programs required to build the intricate subsensory regions, each with a specific nano-scale microarchitecture adapted to a specific sensory need.

### The Functional Anatomy and Physiology of the Five Vestibular Subregions in Mammals

**Figure 2:**
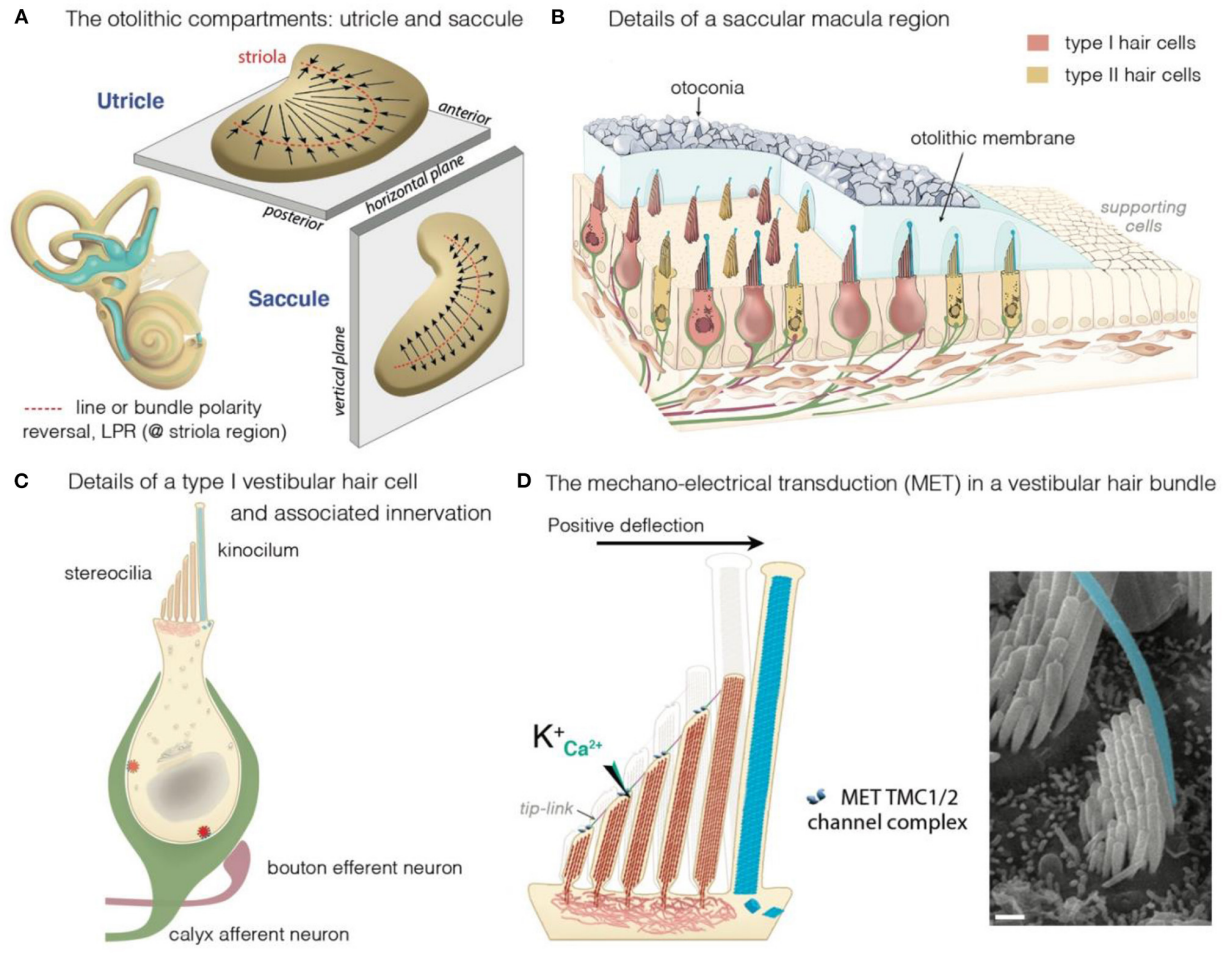
The vestibular maculae, the hair cells and motion-induced mechano-electrical transduction. **(A)** Illustration of the morphological and the polarization of hair cells in the utricular and saccular maculae: the black arrows indicate the orientation of the hair bundles. In the utricle, hair cells are oriented with their stereociliary bundles pointing toward the line of polarity reversal (LPR, in red). In the saccule, the stereociliary bundles point away from the LPR. **(B–D)** Overview of a macular sensory region **(B)**, type I vestibular hair cells (VHCs) **(C)**, and the hair bundle **(D)**. **(B)** The VHCs extend their hair bundles into the overlying otolithic membrane; an extracellular matrix embedded with otoconia. The kinocilium is colored in cyan. Apart from difference in shape, type I and type II hair cells can also differ according to their innervation. Afferent (green) and efferent (dark purple red) are shown. **(B,C)** The VHCs are innervated by three types of afferent neurons: The calyx-only class of neurons makes contacts with type I hair cells, essentially in the striola region; dimorphic neurons contact both types of hair cells throughout the sensory epithelium, forming calyces with type I hair cells and boutons with type II hair cells; bouton-only afferents only contact type II hair cells located outside of the central striola region. **(D)** The basic transduction mechanism is the same in the auditory and vestibular hair cells: a mechanical stimulus bends the stereociliary bundle toward the kinocilium (artificially colored in cyan). The bundle deflection stretches the tip-link, causing an influx of K^+^ and Ca^2+^ ions into the transducing stereocilia. The ensuing change in cell membrane receptor potential leads to glutamate release, generating electrical signals in afferent neurons (green in **B,C**) forming the VIIIth cranial nerve.

The inner ear houses the sensory organ for hearing, the cochlea, and the organ responsible for balance, the vestibule (Figures 1A–C) (25, 39–43). The scala media of the three fluid-filled turns of the cochlea houses the auditory sensory epithelium, the organ of Corti (Figure 1C) (25, 39–43). Five subregions can be distinguished in the mammalian vestibular apparatus: three semicircular canals, and two otolithic organs (Figure 1A) (25, 39–43). The semicircular canals—three tubular structures filled with endolymph—are responsible for dynamic equilibrium, responding to rotational movements (angular accelerations) of the head. The horizontal/lateral, posterior, and anterior/superior canals are oriented orthogonally to each other, each contributing to the sense of balance in a different direction (4, 25, 43, 44). The two otolith organs, the utricle and saccule, account for static equilibrium through their response to different movements of the head (4, 25, 43, 44). They are both essential to the detection of the gravity vector and represent static tilt with respect to gravity. The saccule, which is vertically oriented, responds to linear accelerations in the vertical plane, whereas the utricle, which lies horizontally, detects lateral displacements (Figure 2A).

The mechanosensitive hair cells are located in the crista ampullaris of the semicircular canals (Figures 1A,B), and the maculae of the utricle and saccule (Figures 2A,B) (4, 25, 43, 44). Both organs have type I and type II hair cells, but the otolithic and canal organs have a specific organization (45–48). The stereocilia of crista hair cells are embedded in a glycoprotein-rich gelatinous mass, the cupula, that spans the width of the ampulla, thereby acting as a barrier to endolymph circulation (Figure 1B). The stereocilia of all the hair bundles within the same crista are oriented in the same direction. When the head turns in the plane of one of the semicircular canals, the endolymph in the canal also moves, exerting a force across the cupula. This leads to a deformation of the cupula that is transmitted to the hair bundles of the crista, which are displaced, triggering hair-cell stimulation.

In the maculae of the utricle and saccule, an axis of mirror symmetry in the central region, the striola, divides the organ into two subsets of hair cells with hair bundles of opposite polarizations (4, 25, 43, 44). The stereocilia of the macular hair cells are included in a gelatinous layer, the otolithic membrane, covered by a layer of calcium crystals called the otoconia (Figure 2A). Linear accelerations produce a direct displacement of the otoconia and underlying structures. The otoconia are much heavier than the underlying structures, and this difference in mass causes the otolithic membrane to lag behind the maculae, thereby setting up responses to changes in velocity (acceleration) rather than velocity itself. This shift between the otoconia and the sensory epithelium results in the deflection of the hair bundles embedded in the gelatinous layer, triggering hair-cell stimulation (Figure 2D).

All these hair cells have a mechanosensitive organelle, the hair bundle, at their apical surface [see (49, 50)]. This organelle is the key to our ability to perceive sound and maintain balance (41, 42, 51, 52). Each hair bundle consists of 50–300 F-actin-filled stereocilia, arranged in a highly organized staircase pattern (Figure 2D). A special subset of interstereociliary links, the tip-links, connect the tip of each stereocilium to its adjacent tallest stereocilium and are present in all species (53). These links are essential for the mechano-electrical transduction (MET) process (54–56). The mechanical and geometric characteristics of the hair bundles differ between vestibular hair cells (VHCs), but their morphological and functioning polarity are similar in all vertebrate hair cells. Following hair-bundle deflection (positive direction, toward the tallest row), the increase in tip-link tension causes the MET channel, located at the tip of the stereocilia in all but the tallest rows of the hair bundle, to open (41, 42, 51, 52, 57). This opening of the MET channels results in an inward current, sustained principally by K^+^, but also by Ca^2+^ ions, causing membrane depolarization and regulating the probability of the MET channels opening (54, 55). In VHCs, it ultimately leads to the opening of the voltage-gated calcium channels at the basolateral membrane. The ensuing Ca^2+^ influx triggers the fusion of synaptic vesicles to the plasma membrane, the release of glutamate and, finally, the transmission of a signal to the brain, providing information about motion of the head and its orientation with respect to gravity (Figures 2C,D) (58).

Like the cochlea, the vestibule displays a high degree of architectural organization in all its compartments and subregions, with multiple parameters, including dimension, volume, liquid composition, radius of the canal, and vestibular vs. cochlear chambers, constructed to facilitate optimal responses to species-specific directional (gravity and motion displacements) and acoustic (frequency range) features (29, 44, 59). The construction of this diversity involves strict developmental programs that are finely tuned in time and space, and necessary for adaptation to the specific needs of aquatic, terrestrial and aerial species (1–5). Cross-species comparative morphological and molecular analyses of neurosensory epithelia from distantly related species may provide a wealth of information, highlighting essential organ and cell similarities and differences shedding light on the development and functioning of the inner ear suborgans.

## Evolutionary Changes in the Inner Ear: Structure-Function Correlations and Consequences

### Balance and Hearing During Evolution

**Figure 3:**
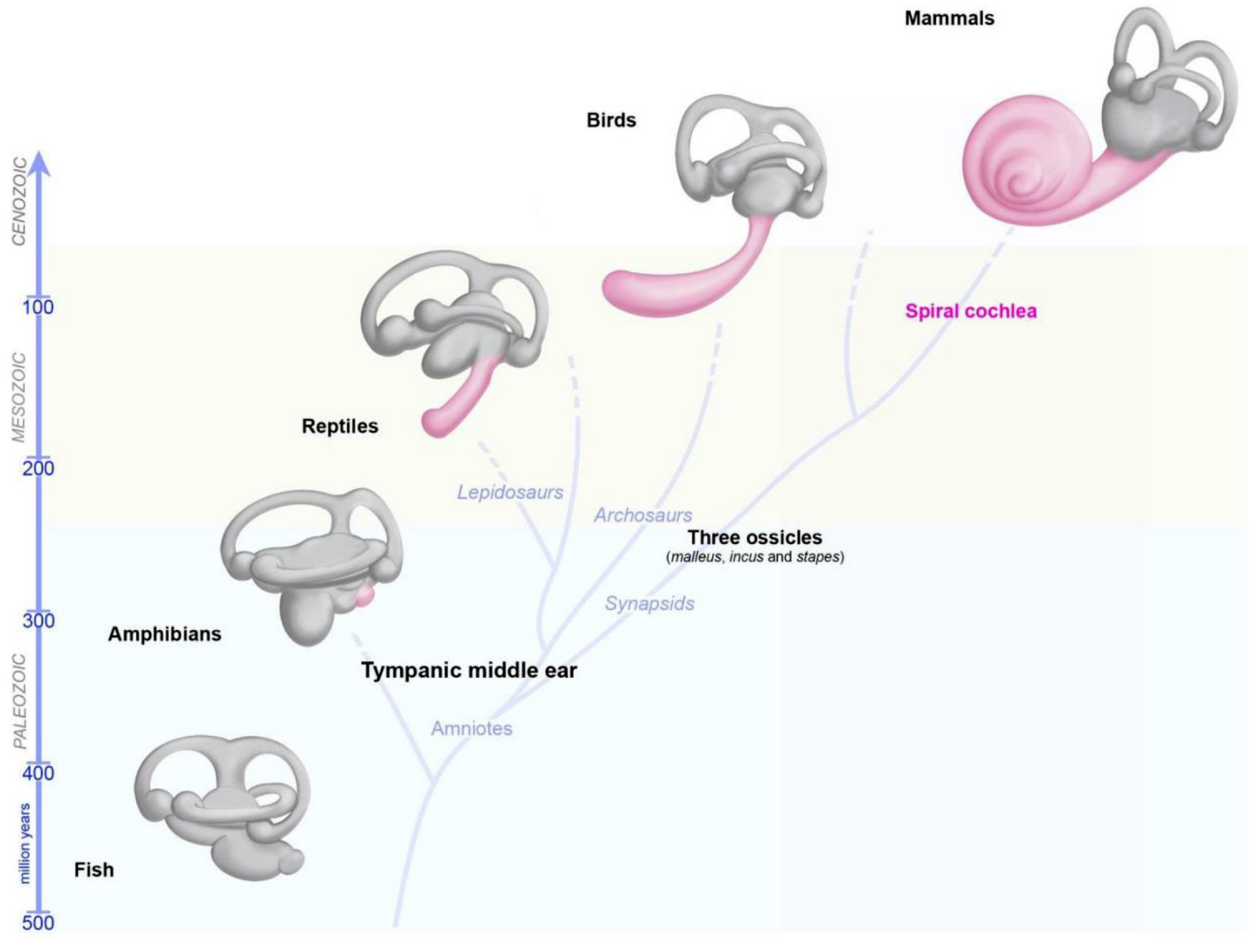
Evolution of the inner ear in vertebrates. The global organization of the five vestibular end organs (utricle, saccule, and the three semicircular canals) has changed very little from fish to current mammals. A sense organ dedicated exclusively to hearing, the papilla, appeared for the first time in amphibians, and then progressively elongates in reptile and birds, and form a spiral in modern mammals.

The capacity of the inner ear to detect various stimuli in different environments has led to studies tracing the precise evolutionary origins of its compartments. Comparative anatomy studies based on fossil records, and cross-species studies, have provided a wealth of information about the role of convergent evolution in creating analogous ears adapted to specific needs in distantly-related taxa (26, 29, 60, 61). Recent reviews have provided detailed information about the development of the inner ear and changes during its evolution, and about the independent evolution of mechanosensitive cells to accommodate mechanical senses (26, 29, 60, 61). Focusing on vertebrates, we highlight here some major changes pertaining to the hearing and balance organs of the inner ear (Figure 3). Initially, at the start of its evolution, the inner ear consisted exclusively of vestibular epithelia (26, 29, 60). The fossils of the earliest jawed fishes, dating from more than 450 million years ago, show that these animals had inner ears consisting of three semicircular canals and the utricular and saccular organs—an organization that persists in their descendants, including sharks, bony fish, amphibians, reptiles, mammals, and birds (26, 29, 60, 61). The cellular organization of the neurosensory epithelium is similar in all vestibular organs, with highly specialized hair cells surrounded by supporting cells, and overlaid by an acellular gelatinous membrane (Figure 2B, mammalian maculae). Fish (which account for half of all living animals) have no dedicated auditory epithelium. They use their vestibular sensory organs (the saccular maculae in most teleost fishes) to perceive acoustic signals (26, 29, 60, 61). An organ exclusively dedicated to hearing, the basilar papilla, first emerged in amphibians about 380 million years ago, when vertebrates moved from an aquatic to a terrestrial environment (Figure 3) (26, 29, 60, 61). The hearing organ subsequently increased in size, leading to improvements in the resolution of sound frequencies for given ranges of species-specific perceived frequencies (26, 29, 60, 61). The spiral shape of the cochlea evolved later in mammals, about 120 million years ago, just before the therians split into the marsupial and placental lineages (Figure 3).

During these evolutionary transitions, pre-existing and new cells and structures appeared and evolved within the inner ear or became associated with it, thereby creating new settings, or further optimizing existing specializations. These major new additions included the appearance of the tympanal middle ear, the basilar and tectorial acellular membranes in the cochlea, the increasing specialization of the supporting cells, hair cells and the associated innervation, and continual changes in the geometry and composition of the hair bundles. In addition to the emergence of two types of hair cell in the vestibular apparatus, one crucial event was the appearance of the outer hair cells in the cochlea, a type of electromotile hair cells found only in mammals.

### Differences in the Distribution, Structure and Function of the Vestibular Hair Cells

In the cochlea, hearing depends on 12,000–15,500 hair cells divided in two types: the outer hair cells (OHCs; 9,000–12,000 cells organized into three rows), which amplify sound stimuli, and the inner hair cells (IHCs; a single row of 3,000–3,500 cells), the genuine sensory cells responsible for transmitting sensory information to the central nervous system. The vestibular sensory epithelia in mammals also contain two types of hair cells—type I and type II—with different morphological, molecular, electrophysiological and synaptic features (Figures 1, 2) (45, 47, 62, 63). As in the cochlea, the hair cells and adjacent supporting cells are sealed together by tight adherens junctions, maintaining a clear separation between the endolymph bathing the apex of the cells, and the perilymph, in which the cell bodies are immersed. Type II hair cells are thought to have evolved first; they occur in all vertebrate vestibular epithelia, whereas type I hair cells are found only in amniotes (reptiles, birds, and mammals) [reviewed in (29)]. The regional distribution of both cell types varies between species; but type I cells are generally most abundant at the apex/center of the cristae ampullaris and in the central striola region of the maculae (47, 63, 64). Each of the sensory cell types is characterized by different anatomical features at the micro- and nano-scales, and these features are associated with functional outcomes in terms of specific mechanical properties, and receptor sensitivities (47, 62, 64–67). The seminal studies performed by Lewis Tilney over the course of a decade on hair bundles in the hearing organ of chickens described the precise sequential steps in hair bundle formation and maturation. According to their position in the chicken basilar papilla, the hair bundles have a specific number of stereocilia, with specific length, width, and arrangements (68–71). However, few studies to date have addressed the precise architecture of vestibular hair bundles in vestibular organs and their variations, or the variation of the position of these hair bundles in a given suborgan. Several groups have shown that the hair bundles of type I hair cells are wider than those of type II hair cells, with taller stereocilia, arranged to form a steeper bundle slope (44, 62, 72, 73). This architecture may translate into type I bundle responses displaying a phase advance relative to those of type II hair cells. Studies of the utricular macula have shown some differences between the hair bundles of type I hair cells in the striola and those in the lateral or medial extrastriolar regions (44, 62, 72). In particular, the striolar hair bundles in vertebrate otoconial organs are stiffer, which may improve the signaling of high head accelerations or high-frequency components of head movements (44, 72).

Type I and type II hair cells also display differences in innervation. The vestibular afferent neurons form three groups on the basis of terminal structure: bouton, pure calyceal (“cup-like” calretinin-positive terminals), and dimorphic (terminating in both calyceal and bouton endings) (Figures 2B,C). The calyceal afferent terminals surround the basolateral surface of one or more “pear-shaped” type I hair cells. Type II cells have a cylindrical body and connect to multiple afferent bouton synapses (Figures 2B,C). Both types of hair cells also receive cholinergic efferent innervation, directly to the soma of type II cells [shown in frog (74)] and via the afferent terminals for both type I and type II cells (Figures 2B,C) (46, 62, 66). Type II hair cells also have thinner and fewer stereocilia per hair bundle (45, 62). The type I hair cells have a relatively hyperpolarized resting membrane potential, −80 mV, dominated by a large, slowly activating K^+^ current (I_K, L_). Their primary current is a large non-activating K^+^ delayed inward rectifier, which allows these cells to produce rapid voltage responses and signal transmission (66). Type II hair cells have a more depolarized membrane potential and a smaller activation range (47, 66, 67). We still have much to learn about how calyceal synapses evolved, but one of the driving forces behind their evolution exclusively within the vestibular system may be the need for faster reflexes in a terrestrial environment (29, 47, 75). Further investigations of the relationships between synaptic features, and the geometric and mechanical properties of the hair bundles are required, to shed light on both phylogenic aspects of evolution and morphometric sets for functional outcomes.

### Structural and Molecular Evolutionary Changes and Hair-Cell Regeneration

As described above, changes in major cellular processes, protein profiles, and interacting regulatory pathways in the hair cells and adjacent cells have been necessary to accommodate the perception of increasingly high frequencies. One of the major consequences of these adaptive morphological and molecular changes was a loss of the ability of hair cells to regenerate (76). Interestingly, the ears of sharks, bony fish, amphibians, reptiles, and birds continue to produce hair cells throughout the life of the animal (77). New hair cells are produced continually as the hair-cell populations turn over, as in fish inner ears, or through the spontaneous replacement of damaged hair cells, as in chickens. In the avian cochlea, many mature supporting cells retain a latent capacity to de-differentiate. Upon hair cell damage or death, these supporting cells re-enter the cell cycle and differentiate to produce new hair cells or supporting cells (78, 79). However, for yet unknown reasons, the mature mammalian hearing organ displays an almost total loss of regenerative capacity. There is no compelling evidence for the existence of a regenerative process in the adult organ of Corti for the production of new, healthy, mature, functional hair cells. Nonetheless, several studies have reported that mammalian ears may harbor some reserve stem cell populations (59), although this “stemness potential” is limited in both time and space. A subset of supporting cells that can develop into new hair cells has been identified, mostly in young animals, before the onset of hearing (27, 59). In adult mammals, some vestibular type II hair cells also still have preserved regenerative abilities. After near complete elimination of all hair cells in *diphtheria toxin receptor* (*DTR*) mice exposed to dyptheria toxin, up to 40% of the type II hair cell population is regenerated, through regional trans-differentiation of supporting cells (80). Recent findings further support this regional segregation of hair-cell precursors with various hair-cell regeneration capacities. Regenerated hair cells derived from Plp1^+^ supporting cells (from the extrastriolar/peripheral region) display type II hair-cell properties. Lgr5^+^ supporting cells in the striolar region may have either type I or II hair-cell properties (32). However, whilst postnatally generated and regenerated hair cells acquire many somatic properties (e.g., morphology, hair-cell subtype markers, synaptic markers, nerve terminals, and basolateral conductance), the structural and functional MET properties of the hair bundle remain immature (32). Further studies are required to overcome these aberrant or incomplete maturation programs, why type I hair cells have less or no preserved regenerative abilities, and to confirm the stability over time and functional activity of regenerated cells and the absence of late degenerative processes after regeneration.

Major efforts are underway to unravel the intricacies of hair-cell regeneration in older mammals, through transcription factor supplementation to reprogram non-sensory inner ear cells to develop into hair cells in particular (31, 34). Regeneration is a tightly regulated, multistep process with high energy demands. Cell de-differentiation and activation of new differentiation cycle pathways trigger major changes in tissue organization, the remodeling of established cell-cell junctions, and a reshaping of proliferating or newly converted cell types. Cross-species comparisons of the mammalian ear and the regeneration-competent ears from non-mammals have highlighted inverse correlations of the ability of the inner ear organ to regenerate with organ complexity and the degree of structural and cellular specialization. The intricate organization of the mammalian hearing organ, with highly specialized hair cells, neurons and supporting cells, is unmatched in the animal kingdom. Studies of vestibular organs have also revealed a massive thickening of the circumferential F-actin belts supporting cell-cell junctions in mouse and human vestibular neurosensory epithelia (81–85). This cytoskeletal reinforcement, which occupies almost 90% of the cell at the intercellular junction, is further strengthened by high levels of adhesion proteins in adult mammals, as shown for the E-cadherin and P120 catenin proteins (81, 82). Conversely, similar studies in five classes of non-mammals revealed a lack of junction reinforcement, with the actin belts in the utricular supporting cells remaining thin, from hatching through adulthood. Cadherin levels at supporting cell junctions remain low (or absent) throughout life in fish, frogs, turtles, and chickens, consistent with the preserved capacity for regeneration in these species (83–85).

Further studies of the regeneration-competent ears of non-mammals, and comparisons between mammalian ears will probably help to identify potential permissive and limiting factors for hair-cell regeneration. These efforts should also address the issue of whether the re-activation of a lost signal in an already established and highly stabilized mature tissue, as in mammals, is feasible without potential late degenerative processes triggering deleterious consequences over time. With the advent of single-cell transcriptomics (scRNA-seq), evidence has been obtained for cell heterogeneity within the vestibular subcompartments (48, 86, 87). There are probably spatially different supporting cell types with different types of regenerative behavior. Careful attention should also be paid to the organization of the whole organ, and the crucial need to re-establish close correlations and connections between hair cells, neurons, and neighboring supporting cells.

## Vestibular Disorders Associated With Hearing Loss: Diversity of Causes/Origins, and Clinical Presentations

**Figure 4:**
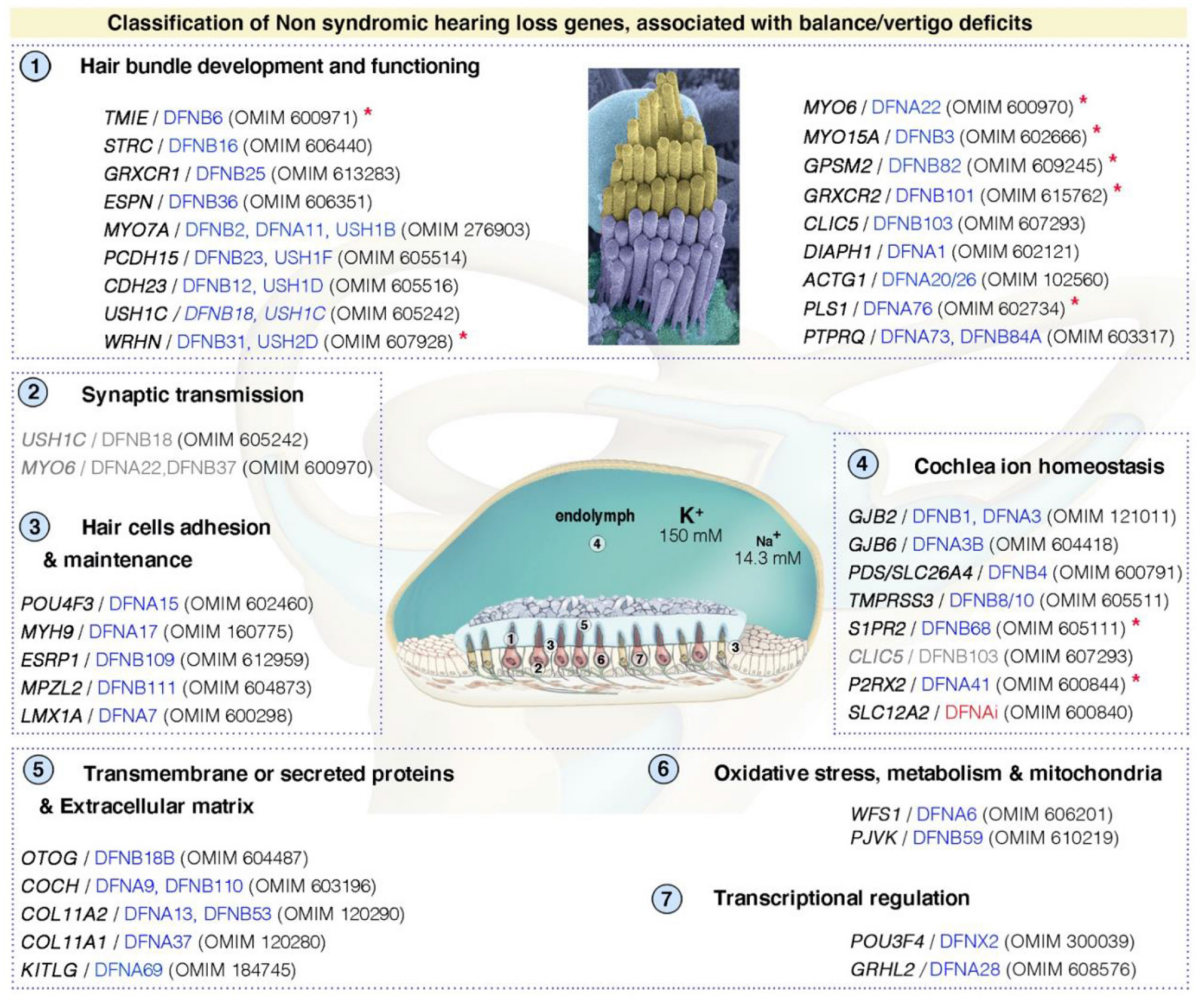
Functional stratification of genes/proteins causing isolated deafness hearing loss, combined with vestibular deficits. Only human genes responsible for non-syndromic deafness, and for which vestibular deficits have been reported based on clinical findings in affected patients (cf. OMIM numbers); and established role and characterization of corresponding animal models, are shown. These can be grouped into several functional categories: (1) hair bundle development and functioning, (2) synaptic transmission, (3) hair cell's adhesion and maintenance, (4) cochlea ion homeostasis, (5) transmembrane or secreted proteins and extracellular matrix, (6) oxidative stress, metabolism and mitochondrial defects, and (7) transcriptional regulation. DFNAi (red) denotes autosomal-dominant forms of deafness with undefined locus number. The genes/loci in gray denote that they share several functional categories. Similar stratification has been observed for deafness genes (49). Red asterisks indicate those included based on the presence of unambiguous vestibular deficits only in mouse models. More detailed information regarding the deafness causative genes is provided in Supplementary Table S1.

Like hearing loss (49, 88), vestibular disorders may have multiple causes, including genetics, aging, drugs, immune and environmental factors (e.g., infections, noise exposure) (89). The prevalence of vestibular impairment is high in children and adults with sensorineural hearing loss (SNHL), between 20 and 70% (90, 91). It has been shown that 50% of children eligible for cochlear implantation have some sort of vestibular dysfunction, with up to 20% presenting complete bilateral vestibular impairment (92). Despite the frequent association of vestibular dysfunction with hearing loss, it remains extremely difficult to predict the type or severity of impairment from the characteristics of hearing loss (92). However, the etiology of the hearing loss seems to play a role, and abnormal vestibular responses are more frequently found in specific types of hearing loss (Figure 4; Supplementary Table S1).

A multitude of clinical signs, such as spontaneous nystagmus, whirling vertigo, delayed postural and motor development, recurrent unexplained falling, poor balance on uneven ground or in situations of limited vision and oscillopsia (blurring of the vision with head movement) are indicative of vestibular involvement and should lead to a detailed evaluation of vestibular function. In case of oscillopsia or delayed postural and motor development bilateral vestibular loss should always be eliminated. However, some of these clinical signs (e.g., whirling vertigo, spontaneous nystagmus) may go unnoticed or be absent if both inner ears are damaged simultaneously (as in cases of systemic exposure to a vestibular toxin), if the damage is congenital or if it occurs in young children, as they tolerate dizziness better than adolescents and adults. For this reason, systematic vestibular testing should always be proposed if hearing loss is diagnosed. Complete vestibular assessment is easy to perform in clinical practice (even in young children under the age of 1 year) and includes an evaluation of canal function and otolithic function.

We briefly review here the different exploratory tests for vestibular function, and the vestibular dysfunctions observed in some forms of hearing loss.

### Vestibular Exploratory Tests

Current clinical tests of vestibular function do not directly assess inner ear function. Instead, they evaluate the final motor output of the eyes or balance control systems. *Five* main sets of measurements are routinely recorded in clinics to evaluate the function of the vestibular organs and to determine the pattern specificities of vestibular disorders: c-VEMP, o-VEMP, caloric tests, rotatory chair test and head impulse tests (HITs) (92–94). cVEMP is recorded for the sternocleidomastoid muscle (SCM). This potential is a manifestation of the vestibulospinal reflex and saccular function. oVEMP is recorded for the extra-ocular muscles and is a manifestation of the VOR and utricular function. Canal function can be evaluated at 3 head velocities: at low frequency with bithermal caloric test (0.003 Hz); at middle frequency with rotatory tests (0.01–0.5 Hz) and at high frequency with the head impulse test (5–6 Hz). In the caloric test, warm and cooled water or air in the ear canal is used to stimulate the horizontal semicircular canal, causing nystagmus, which can be monitored by video tracking. The use of a rotatory chair with monitoring of the eye movement allows for the evaluation of the horizontal semicircular canal function at middle frequency. When tilting the axis of the chair rotation with respect to the gravitational axis, the otolith function can be tested (OVAR- off axis vertical testing). Head impulse testing can be used to test the function of all three semicircular canals at high frequency.

Compensatory mechanisms often occur in the central nervous system, with the re-adjustment of neural circuits and centers to overcome decreased input from the peripheral vestibular system. Vestibular assessments are not often included in routine phenotyping in clinics. Vestibular impairments present at birth, and moderate or progressive deficits may therefore be overlooked or undetectable due to the indirect nature of the information collected on questionnaires or through behavioral observations. It is important to note that even if compensatory mechanisms frequently occur, in particular by using vision and proprioception inputs, not all compensation strategies are adequate and in case of complete vestibular loss some functions can never be entirely compensated (e.g., gaze stabilization during movement). Moreover, all compensation mechanisms come with a cost in terms of attention and fatigue.

Animal models provide opportunities to obtain additional, more detailed information about vestibular functions, even in the absence of overt balance deficits (95–98). Behavioral tests in animals include the monitoring of behaviors suggestive of balance problems (e.g., head bobbing, wobbling, circling behavior, spatial misorientation, reaching and air-righting reflexes, gait, swimming tests, beam-crossing, platform or rotarod assays) (99). The recording of eye movements, such as the angular VOR (aVOR) and optokinetic response (OKR), have also been used to provide a more complete analysis of peripheral vestibular function (100, 101). If the OKR is preserved, the VOR deficits detected can be used to infer a peripheral abnormality, such as semicircular canal function (vestibular afferent response to earth-vertical axis head rotation, EVAR) or otolithic function (vestibular afferent response to off-vertical axis head rotation, OVAR). These eye movement recordings also reflect the input, integration and output components of nervous system function. Direct measurements of peripheral vestibular function through invasive tests can be performed on animals. For example, vestibular compound action potentials (also known as vestibular evoked potentials, VsEP) can be recorded from individual neural elements in animals. They reflect the direct activity of the vestibular nerve and central relays in response to linear head acceleration (102). Their measurement provides comparative values of response thresholds, peak latencies, and peak-to-peak amplitudes, and more precise indications of the sensitivity of the gravity receptor end organs, the utricle and the saccule (102, 103). Based on these animal research, the development of similar electrophysiological measurement of peripheral vestibular function has been initiated in humans (104). Such extensive, in-depth research on animal models is essential to improve our understanding of the correlation between vestibular symptoms and disease etiology and to facilitate the accurate evaluation of treatment strategies for protecting or restoring vestibular function (95–98, 105).

### Sources of Hearing and Balance Deficits

#### Viral Infection

Congenital cytomegalovirus (CMV) infection is the main non-genetic cause of SNHL, implicated in 8% of cases of hearing loss of any degree of severity (106). It is estimated to be responsible for 20% of cases of hearing loss of unknown origin. Vestibular dysfunction is particularly frequent in patients with congenital CMV infection. A French retrospective study of auditory and vestibular assessments on 52 children with congenital CMV infection (107) showed that more than 90% of children with CMV-related hearing loss had a vestibular impairment and that a third of these children had a complete and bilateral loss of vestibular function (vestibular areflexia). However, no correlation was found between the severity of hearing loss and that of vestibular dysfunction. The pathogenetic hypothesis, derived from fetal and animal data, concerns the potassium regulation structures of the endolymphatic sector of the inner ear (108): the cochlear stria vascularis and vestibular dark cells, both of which are prime targets of CMV. Endolymphatic potential is essential for auditory sensory cell depolarization; lesions of the cochlear stria vascularis and vestibular dark cells may impair potassium recycling, thereby decreasing endolymphatic potential (109). Paradoxically, hair cells seem to be spared by viral infection. Hearing loss and vestibular disorders may therefore be due to a deregulation of potassium flow, with progressive decrease of endolymphatic potential explaining a delayed onset of hearing loss or a fluctuation of hearing loss with time.

#### Bacterial Infection

Hearing loss is the most common complication of bacterial meningitis, affecting more than 50% of survivors of pneumococcal meningitis (110). Meningitis can also cause a loss of vestibular system function, potentially via the same pathophysiological mechanisms, which are assumed to involve bacterial invasion of the inner ear followed by inflammatory infiltration. Vestibular dysfunction is frequently associated with bacterial meningitis and can be severe, with up to 14% of patients developing vestibular areflexia in one or both ears (21, 111). In a population of patients with bilateral vestibulopathy, the etiology was reported to be meningitis in 5% of cases (112). A loss of vestibular function due to bacterial meningitis in young children may delay posturomotor development if meningitis occurs before the child begins to walk independently, even in the absence of neurological impairment (21).

#### Noise-Induced Hearing Loss

Vestibular responses can be elicited by sound stimulation, and it is known that the otolith organs are the most sound-sensitive. There is increasing evidence that excessive noise can induce vestibular deficiency by damaging the VHC hair bundle and/or the vestibulo-spinal, VOR pathway (113, 114), the observed hearing threshold being related to VEMP responses (115). Two mechanisms are involved in the destruction of the end organs by noise: direct mechanical injury and metabolic damage to the organ of Corti leading to the degeneration of sensory elements. Nevertheless, clinical studies have reported a low incidence of clinical signs of vestibular malfunction in patients with noise-induced hearing loss (NIHL), probably because of the redundancy of the vestibular peripheral system and compensatory mechanisms (114). However, these compensatory mechanisms may weaken with aging and vestibular dysfunction may therefore become increasingly common with age in NIHL patients (114).

#### Aging

Presbycusis (age-related hearing loss) and presbystasis (age-related changes in the vestibular organ) are becoming increasingly frequent, particularly in developed countries, due to increasing life expectancy. These two systems may age at different rates in the same individual (116), but presbycusis and presbystasis have a number of mechanisms in common, including a dysfunction of inner ear components (e.g., hair cells, ganglion neurons, vascular cells) and age-related changes in the central nervous system (117, 118). It was suggested several years ago that presbycusis and presbystasis have a genetic basis, and genes potentially involved in the pathophysiology of both conditions have been identified (118).

#### Auto-Immune Disorders

SNHL is the most common audiovestibular symptom associated with systemic autoimmune diseases, followed by tinnitus and vertigo (119). The pathophysiology of inner ear involvement in autoimmune diseases remains unclear and may be related to circulating antibodies against several inner ear antigens (primary autoimmune inner ear diseases) or immune complex-mediated damage (inner ear involvement in systemic autoimmune diseases) (120). Immune complex deposition seems to be the major factor involved in cochlear and vestibular damage in systemic autoimmune diseases. The deposition of immune complexes reduces the caliber of the auditory arteries, leading to a decrease in blood flow, inducing an oxygen deficit and increase in the levels of reactive oxygen species responsible for damage to hair cells and spiral ganglion (121). The vestibular symptoms observed in systemic autoimmune diseases may be isolated, or part of a spectrum, as in Meniere's disease. However, many patients have asymptomatic vestibular involvement requiring an objective vestibular assessment. Treatment may be based on systemic steroids, antimetabolites, such as methotrexate, or intratympanic steroid treatment in some cases.

#### Ototoxic Drugs

Drugs toxic to the vestibule are frequently used, especially in patients with serious infections or life-threatening tumors (122, 123), see also ^1^. Vestibulotoxicity can affect hair cells in the utricle, saccule, or semicircular canals, leading to diverse vestibular deficits^2^. The risk of developing a vestibular deficit depends principally on the dose and properties of the toxic drug, probably modulated by genetic predisposition. Some of the drugs that damage the vestibule also harm the cochlea and, therefore, also cause hearing loss and/or tinnitus, but others have toxic effects specific to the vestibular system, and vestibular dysfunction may be more difficult to diagnose. The main substances responsible for significant vestibular toxicity are antibiotics, particularly aminoglycosides, and chemotherapy agents, such as cisplatin. Aminoglycosides, especially streptomycin, neomycin and gentamicin, are the most toxic drugs to the inner ear and their vestibulotoxicity is even greater than their toxicity to the cochlea. For the vestibular system, there is no safe dose of gentamicin and no safe serum gentamicin concentration (124). The use of aminoglycosides can result in a dramatic loss of VHCs (123). At the subcellular level, these drugs induce changes including the hydropic and vacuolar degeneration of VHCs (125).

Cisplatin is a widely used anticancer agent with toxic effects on the sensory epithelia in the vestibular and cochlear systems. It has been reported not only to induce a marked decrease in hair cell density in the utricle, saccule, and ampullae, but also to result in an abnormal morphology and disorganization of the VHCs and their stereocilia bundles (126). Other substances, such as organic solvents, have toxic effects on the parts of the brain responsible for processing balance information, rather than on the vestibular system itself (127).

#### Hereditary Hearing Loss

By performing a literature survey, including studies of the available mutant mice, along with the HHH Hereditary Hearing Loss database [https://hereditaryhearingloss.org, (128)] and the OMIM (Online Mendelian Inheritance in Man; https://www.omim.org/) database, we defined a list of genes for which defects lead to vestibular dysfunction (Figure 4; Supplementary Table S1). *DFNA, DFNB, and DFNX* denote isolated forms of human deafness with autosomal dominant (DFNA), recessive (DFNB) or X-linked (DFNX) transmission (128). Vestibular dysfunction can be suspected in 30% of the listed non-syndromic forms of SNHL (Supplementary Table S1). In fact, the prevalence of associated vestibular dysfunction seems to be higher for syndromic hearing loss (Supplementary Table S1). Indeed, the prevalence of vestibular dysfunction is probably underestimated because many studies fail to detect such dysfunctions due to their focus on other sensory modalities or the inherent difficulty of objective direct assessments of vestibular function. However, as mentioned above, tools for the clinical assessment of vestibular function are available and can provide a considerable amount of information about peripheral vestibular defects in patients with hearing loss. Additional studies are required, focusing on determining the real spectrum of vestibular dysfunction in hearing loss patients. Vestibular testing should be included as an integral part of all hearing loss evaluations. Some efforts have been made in this direction. For example, Belgium has implemented a vestibular defect screening test for children with hearing impairment (129) and the French Society for Otorhinolaryngology (SFORL) recommends a vestibular assessment for all children undergoing cochlear implantation in its latest guidelines on pediatric cochlear implantation (130).

**Table 1:** Gene therapies in animal models with vestibular deficits.

**Protein gene name deafness locus or disorder**	**Animal model *Delivery route (age at delivery)***	**Vector/treatment & therapy outcomes in vestibular end-organs**	**References**
Harmonin *USH1C* DFNB18	Ush1c c.216G>A knockin 216AA mice *IP injection (P3, P5, P10, P13, P15 and adult)*	***ASO-29*** - Correctly spliced Ush1c mRNA and increased harmonin protein expression. - Rescued balance behavior (open field^*^, circling^*^, swimming^*^, trunk-curl^*^ contact-righting^*^) after injections before P15. *- Oldest testing point: 9 months*.	(134)
	Ush1c c.216G>A knockin mice *IP injection (P3, P4, P5, P15, P1/3/5/7)*	***ASO-29*** - Normal balance behavior (circling^*^, swimming^*^) and vestibular function (VsEP^*^) after neonatal injections. *- Oldest testing point: P30*.	(135)
	Ush1c c.216G>A knockin mice *IP injection (P5)*	***ASO-29*** - Improved spatial orientation (exploratory movements). - *Oldest testing point: 6 months*.	(136)
	Ush1c c.216G>A KI *RWM injection (P1) PSCC injection (P1) Topical TM application (P5) Trans-TM injection (P10 and P20)*	***ASO-29*** - Restored vestibular function (rotational VOR, irregular VAR) after PSCC injection. - Improved balance behavior (circling^*^) after topical-TM, RWM and trans-TM delivery. *- Oldest testing point: 2 years*.	(137)
	Ush1c c.216G>A knockin mice *Intra-otic injection (E12.5)*	***ASO-29*** - Rescued balance behavior (open-field^*^, circling^*^, rotarod^*^, swimming^*^, reaching reflex^*^). *- Oldest testing point: 6 months*.	(138)
	Ush1c c.216G>A knockin mice *RWM injection (P0-P1 and P10-12)*	**AAV2/1-CMV>*****dtTomato::harmonin-a1*** **AAV2/1-CMV>*****EGFP::harmonin-b1*** **AAV2/Anc80L65-CMV>*****harmonin-a1*** **AAV2/Anc80L65-CMV>*****harmonin-b1*** - EGFP::harmonin-b1 detected at the tip of VHC stereocilia. - Rescued balance behavior (rotarod, open field^*^, circling^*^) only after neonatal delivery of exogenous harmonin-a1 and/or b1. *- Oldest testing point: P42*.	(139)
KCNE1 Jervell and Lange-Nielsen syndrome type 2 (JNLS2)	Kcne1^−/−^ mice (129-Kcne1^tm1Sfh^/J) *PSCC injection (P0-P2)*	***AAV1-CB7>Kcne*** - Preserved morphologies of VHC ciliary bundles. - Improved balance behavior (head and gait stability, circling^*^, rotarod^*^, swimming, birth and litter survival rates). - No hearing preservation without vestibular improvement. *- Oldest testing point: 6 months*.	(140)
LHFPL5 DFNB66/67	Lhfpl5^−/−^ mice *RWM injection (P1-P2) Cochleostomy (P0-P1)*	***Exo-AAV1-CBA>HA-Lhfpl5 Exo-AAV1-CBA>HA-Lhfpl5-IRES-GFP*** - Vector diffusion in vestibular sensory epithelia. - Improved balance behavior (head tossing, circling). *- Oldest testing point: P42*.	(141)
Pendrin SLC26A4 DFNB4 and Pendred syndrome	Slc26a4^−/−^ and Slc26a4^tm1Dontuh/tm1Dontuh^ *Otocyst transuterin injection (E12.5) Endolymphatic sac (E12.5)*	**rAAV2/1-CMV>*****Slc26a4-turboGFP*** - Prevention of the membranous vestibular labyrinth enlargement, but no otoconia formation and no recovery of balance behavior (rotarod). *- Oldest testing point: P35*.	(142)
	Slc26a4^−/−^ mice *Otocyst transuterin electroporation (E12.5)*	**CMV>*****Slc26a4-EGFP*** **plasmid** - Rescued balance behavior (open field^*^, circling^*^, swimming). *- Oldest testing point: P30*.	(143)
Sans USH1G	Ush1g^−/−^ mice *RWM injection (P2.5)*	**AAV2/8-CAG>*****Sans*****:IRES:*****GFP*** - Robust transduction of VHC and restored morphology of VHC bundles. - Restoration of balance behavior (open field^*^, circling^*^, swimming^*^, elevated platform, trunk-curl^*^, contact-righting) and vestibular function (angular VOR, OVAR VOR, MOR). *- Oldest testing point: 1 year*.	(144)
Tmc1; DFNB7/11 Tmc1Bth/+; DFNA36	Tmc1^delta/delta^;Tmc2^delta/delta^ mice *RWM injection (P0-P2, P4, P7, P14, P30)*	**AAV2/Anc80L65-CMV>*****Tmc1*****:WPRE AAV2/Anc80L65-CMV>*****Tmc2*****:WPRE AAV2/Anc80L65-CMV>*****Tmc1EX1*****:WPRE** - Neonatal injection of AAV-Tmc2 improves balance behavior (circling^*^, rotarod) and rescues vestibular function (rotational VOR^*^, linear VOR). - AAV-Tmc1 or Tmc2 both improve rotarod performances. - Mice injected with sAAV-Tmc1 were better breeders. *- Oldest testing point: P56*.	(145)
Whirlin (Whrn) USH2D DFNB31	Whirler (Whrn^wi/wi^) mice *PSCC injection (P4)*	***AAV8-CMV>Whrn*** - Robust Whirlin expression in utricular VHC, at the stereocilia tips. - Morphological restoration of utricular VHC (stereocilia length). - Improved balance behavior (open field, circling, swimming, rotarod). Improved VsEPs but only for bilateraly injected mice. *- Oldest testing point: 4 months*.	(146)
Ototoxicity	wt C57BL6 mice + AMG-induced ototoxicity *Cochleostomy (scala tympany) (adult)*	***Ad-CMV>Math1.11D*** - Restored vestibular epithelium thickness. - Preservation of VHC in saccular and utriclar maculae and cristae. - Improved balance behavior (swimming) and vestibular function (horizontal VOR). *- Oldest testing point: 8 weeks post-injection*.	(147)
	wt C57BL/6 mice + IDPN-induced vestibulotoxicity *RWM injection (adult)*	***Ad28-GFAP>Atoh1*** - Restoration of utricular and saccular VHC populations. - Rescued balance function (rotarod). *- Oldest testing point: 2 months post-injection*.	(148)
	wt C57BL/6 mice + AMG-induced ototoxicity *PSCC (adult)*	***Ad5-hCM>Bcl2*** - Preservation of VHC and stereociliary bundles.	(149)
	wt CD1 mice + AMG-induced ototoxicity *PSCC (adult)*	***Hes5 siRNA*** - Increased number of utricular VHC.	(150)
	Rat + AMG-induced ototoxicity *Scala vestibuli* (adult)	***Ad-Math1-EGFP*** - Regeneration of type I VHC and synaptic formation.	(151)
	Guinea pigs + AMG-induced ototoxicity *Cochleostomy (scala vestibuli) (adult)*	**Ad-*****hGDNF*** - Preservation of utricular VHC and stereociliary bundles.	(152)

* *comparable to age-matched unaffected individuals*.

*AAV, adeno-associated viral vector; Ad, adenoviral vector; AMG, aminoglycoside; ASO-29, antisense oligonucleotide blocking the 216A cryptic splicing of Ush1c mRNA; DFNA, autosomal dominant non-syndromic forms of deafness; DFNB, autosomal recessive non-syndromic forms of deafness; Exo-AAV, exosome-associated AAV; IDPN, 3,3'-iminodipropionitrile; IP, intra-peritoneal; MOR, macula-ocular reflexes; OVAR, vestibular afferent response to off-vertical axis rotation; PSCC, posterior semi-circular canal; RWM, round window membrane; SC, supporting cells; siRNA, small interfering RNA; TM, tympanic membrane; VAR, vestibular afferent response to head rotation; VHC, vestibular hair cell; VsEP, vestibular sensory evoked potential; VOR, vestibulo-ocular reflex; wt, wild-type [Table adapted from Table 1, (49)]*.

## Detailed Mechanisms of Vestibular Function: Insight From Animal Models

Our knowledge of the development and function of the vestibular sensory system owes much to experimental approaches with various vertebrate species. Standard clinical applications, such as the testing of VOR by head impulses or the quantification of otolith function by VEMP, are based on evidence gained from experiments in various animal models (97). Insight into the pathogenic mechanisms at the origin of hearing loss, sometimes associated with vestibular dysfunctions, has also been obtained through the study of animal models of genetic deficits underlying deafness in humans (95, 96, 131, 132). About 140 genes responsible for non-syndromic forms of deafness in humans and about 200 genes responsible for syndromic forms (defects of the inner ear and other organs, including in mice) have been reported (133). Mouse models exist for most of these human genes. A high degree of conservation of anatomical and functional organization has been reported between rodents and humans (95–98). As a result, the available mutant mice, most of which also display vestibular deficits (Table 1; Supplementary Table S1), have provided valuable information about the key pathways leading to vestibular dysfunction. In studies of organ patterning or MET, the use of more distantly related animal models, such as zebrafish, *Drosophila*, or *C. elegans*, has facilitated the identification of key conserved core biological features and highlighted species-specific features (88, 153–157).

Side-by-side comparisons in animal models have yielded major insight into the molecular, cellular and physiological underpinnings of human deafness and vestibulopathies. Figure 4 depicts some of the causal pathways involved in vestibular dysfunction, based on studies in animal models defective for genes associated with deafness in humans. As reported for hearing (49), the causal genes for vestibular dysfunction can be grouped into several categories affecting: (1) hair bundle development and functioning, (2) synaptic transmission, (3) hair cell adhesion and maintenance, (4) cochlea ion homeostasis, (5) transmembrane or secreted proteins and the extracellular matrix, (6) oxidative stress, metabolism, and mitochondrial defects, and (7) transcriptional regulation. Additional genes implicated in vestibular function are indicated in Table 1, based on clinical observations, and information from related mouse models [see also (103)]. The principal representative examples are described briefly below, highlighting the key roles of the hair bundle (Usher proteins and MET components), ion homeostasis (e.g., cochlin, pendrin), and acellular membranes (e.g., tectorins).

### Structural and Functional Defects of the Mechanosensitive Hair Bundles

The vestibular and auditory hair cells have several anatomical and functional properties in common, due in particular, to the presence of a mechanosensitive hair bundle at their apical surface (Figures 1, 3). Unsurprisingly, therefore, defects of key structural and functional proteins of the hair bundle have been found to cause both hearing and balance deficits (Figure 4; Table 1). Here, we briefly describe some of these molecular and functional pathways affecting the links between stereocilia, the cytoskeleton, and the MET machinery.

Extensive studies on the pathophysiology of the Usher Syndrome, the major cause of combined vision and hearing loss, have provide valuable insight into the formation and functioning of hair bundles. Five main Usher type I genes have been identified, encoding the actin-based motor myosin VIIa (USH1B), the PDZ domain-containing submembrane protein harmonin (USH1C), the scaffold protein sans (USH1G), and two Ca^2+^-dependent adhesion proteins, cadherin-23 (USH1D) and protocadherin-15 (USH1F) (158, 159). All the available USH1 mutant mice faithfully reproduce the profound congenital deafness and bilateral vestibular dysfunction observed in human patients, as demonstrated by their typical circling behavior and the absence of VsEPs in USH1 mice. Studies of these mice, the properties of USH1 proteins and their interactions revealed key roles of this network in the hair bundle (158, 159). Together, cadherin-23 and protocadherin-15 make up the embryonic lateral USH1 links between stereocilia and the links between stereocilia and the kinocilium essential to the integrity of the early developing hair bundles (158, 159). These two cadherins also form the upper and lower parts, respectively, of the tip-link (160), the apical link that gates the MET channels. Harmonin and sans are scaffold proteins essential for the anchoring of the cadherin links to the stereocilia actin filaments. Myosin VIIa is also required for the transport of key hair bundle components (e.g., harmonin and protocadherin-15) into the stereocilia (158–162).

Usherin/USH2A- and Adgvr1/USH2C-deficient mice, which have defects of USH2 proteins, have no vestibular deficits, consistent with clinical observations in most human USH2A and USH2C patients (158, 159, 163). This suggests that the ankle links, fibrous links connecting the stereocilia at their base, are probably dispensable for hair bundle function in VHCs (163–165). Interestingly, whirlin, a PDZ scaffold protein similar to harmonin/USH1C, has been associated with DFNB31 isolated hearing loss (166) and Usher syndrome type 2D (167). Whirlin is present as two distinct isoforms, the long and short variants (WHRN-L and WHRN-S), and it has been shown that, in addition to contributing to the formation of ankle links, this protein is a major factor determining the height and staircase organization of the stereocilia (168–170). Whirlin colocalizes and interacts with two other causal proteins for deafness, MYO15A (DFNB3) and EPS8 (DFNB102), forming a tripartite complex that controls actin polymerization and elongation of the actin core at the tips of the stereocilia (168–170). An absence of both whirlin isoforms causes abnormally short stereocilia and profound deafness and vestibular dysfunction (166, 171). However, WHRN-S expression is sufficient to maintain stereociliary bundle morphology and function in a subset of hair cells, resulting in some auditory responses, but no overt vestibular dysfunction (171, 172). The occurrence of vestibular dysfunction in some USH2 patients warrants further investigation. Indeed, defective vestibular responses were reported in eight of eleven USH2 patients tested in one study, but the lack of a clear genetic diagnosis for these patients precluded the identification of a direct causal role of USH2 (171, 173).

In addition to late-onset and progressive vision and hearing loss, some USH3A patients also develop vestibular dysfunction with various patterns of onset, progression, and severity (159, 174). Defects of the USH3A protein, clarin-1, in mice have also been shown to cause variable vestibular deficits that manifest later in life (175). The precise role of clarin-1 in the MET complex and the origin of the variability of vestibular symptoms remain to be established (176, 177).

Defects in another protein, the phosphatidylinositol phosphatase (PTPRQ), has been shown to affect vestibular function differently in humans and mice. PTPRQ, which is involved in DFNB84A and DFNA73 (128), is a component of the shaft links between stereocilia (178). PTPRQ-deficient mice have no overt behavioral signs of vestibular deficits, but detailed analyses have revealed subtle changes in swimming behavior associated with severe-to-profound macular receptor dysfunction, as confirmed by VsEP responses, and significant stereociliary bundle defects in the vestibular maculae (179). Like PTPRQ, other products of key deafness genes are located at the base of the stereocilia, where the shaft gradually narrows to form a conical taper, serving as the pivot point for deflection of the hair bundle upon stimulation. This includes myosin VI (MYO6/DFNA22), radixin (RDX), taperin (TPRN), chloride intracellular channel 5 (CLIC5/DFNB103) (103, 180, 181). VHCs of mutant mice lacking any one of these proteins display fused, thickened, and elongated stereocilia, accounting for the vestibular phenotype described (103, 180, 181).

The normal development and maintenance of stereociliary actin filaments is the key to hair cell function. Espin (ESPN/DFNB36), plastin-1 (PLS1/DFNA76) and fascin-2 (FSCN2) are three key actin filament crosslinkers that cooperate to produce the composite properties of the actin paracrystalline cores of the stereocilia (181). Espin is the major actin crosslinker, as mutant mice lacking this protein display typical abnormal circling behavior, probably due to a reduction of stereocilium diameter and length in both the auditory and vestibular hair cells (103, 181). By contrast, PLS1- and FSCN2-deficient mice displayed no overt behavioral traits, but the absence of either protein affected vestibular function and stereocilium structure, albeit with a markedly milder phenotype (181).

### Defects of Components of the MET Channel Complex

TMC1 and TMC2 have recently been identified as the core proteins of the MET channel complex, constituting the long-sought vertebrate MET channel subunits (182–185). The correct targeting of TMC1/2 pore-forming MET channel subunits to the tips of stereocilia (57) and their functional integrity involve key interactions with several other essential proteins (184, 185). In addition to the USH1 proteins described above, the lipoma HMGIC fusion partner-like 5 (LHFPL5, also known as TMHS, DFNB67), the transmembrane inner ear (TMIE, DFNB6), and the calcium and integrin-binding family member 2 (CIB2) proteins have been identified as components of the MET machinery at the tips of the transducing stereocilia in auditory and vestibular hair cells (186–189). Consistently, hair cells deficient for most of these proteins present a severe-to-complete loss of MET currents, leading to deafness and vestibular dysfunction (188, 190, 191). Of note, the lack of CIB2 causes profound hearing loss, but no signs of vestibular dysfunction have been observed (192–195), probably due to compensatory mechanisms involving CIB3 or other members of the same protein family (188, 196).

### Defective Ionic Composition of the Vestibular and Endolymphatic Sac Environments

Mutations of *SLC26A4* cause isolated DFNB4 hearing loss or a combination of congenital hearing loss, balance, and thyroid disorders known as Pendred syndrome (197, 198). *SLC26A4* encodes pendrin, a CI/HCO_3_ anion exchanger crucial for fluid homeostasis in the inner ear (197, 199). Studies of *Slc26a4*-insufficient or -null mice have reported a clear disruption of sodium chloride and fluid absorption by the epithelial cells of the endolymphatic sac in these mice (198, 199). The impaired absorption of luminal Na^+^, Cl^−^ and water leads to a swelling of the endolymphatic space, a feature also characteristic of a group of human inner ear malformations known as enlargement of vestibular aqueduct (EVA) disorders, and observed in clinical contexts such as Meniere's disease, experimental endolymphatic hydrops, and space motion sickness (198). While the etiology of abovementioned clinical entities is different, and the precise pathogenic link between the endolymphatic sac and inner ear dysfunction is unknown, alterations to pH, ionic composition, size, or the osmotic pressure of endolymph might constitute a common pathogenic pathway. Knock-in mouse models are being developed for studies of fluctuating EVA phenotypes, and to improve the management of patients with *SLC26A4* defects. A panel of behavioral tests was recently used to document vestibular dysfunction in *Slc*26*a*4^loop/loop^ mutant mice (200). These mice harbor a missense mutation (S408F) resulting in profound hearing loss, but they display various degrees of abnormality in vestibular behavior. The inner ears of all mutant mice with severe vestibular dysfunction display a pathological displacement of the otoconia into the crista ampullaris (200). This genetic predisposition for ectopic otoconia provides an excellent experimental model for studies of the pathophysiology of and possible treatment for benign paroxysmal positional vertigo (BPPV), a relatively common disorder in which the otoconia become detached and lodge in one of the semicircular canals (canalithiasis) or cupulae (cupulolithiasis).

### Formation and Maintenance of the Otoconia and Vestibular Acellular Structures

No mutation of an otoconial component gene has ever been identified in humans. However, based on clinical symptoms in patients and studies in mouse, these structures seem clearly important for normal vestibular function. The prevalence of otoconium-related balance disorders is certainly largely underestimated. The morphology and composition of otoconia can be altered due to genetic and environmental factors, aging, ototoxic drugs, and head trauma (201). BPPV and aging are both high-risk factors for an abnormal dislocation of otoconia, causing an aberrant or ectopic distribution of otoconial debris, leading to dizziness or imbalance.

It is important to understand how otoconia are formed and how their attachment to macular acellular membranes is maintained if we wish to develop ways of influencing their regrowth/regeneration for the treatment of abnormalities. The structure and organization of the otoconia differ between mammals and fish, and even within vertebrates generally. Mammalian otoconia are unique in being the only calcium carbonate-containing biomineral found in normal healthy organs. The barrel-shaped calcitic crystals are partially embedded in the underlying gelatinous acellular membrane covering the neurosensory epithelium in the utricular and saccular maculae (see Figure 3). During head movement, the otoconia, which have a higher density than the surrounding endolymph, move relative to the sensory epithelium, leading to a sensing of either linear acceleration or the gravitational field (which is critical for spatial orientation and balance). In addition, by serving as a reservoir of ions, the otoconia may be involved in maintaining the homeostasis of the utricular and saccular endolymph, and the spatial and temporal gradients of calcium and other ions around the apical mechanosensitive hair bundles. Electrophysiological and behavioral studies have shown that the density of the crystals affects the magnitude of stimulus input to the sensory hair cells and that significant changes in crystal mass or location invariably leads to behavioral deficits.

Multiple, diverse pathways act in concert to ensure the normal biogenesis and maintenance of the otoconia. These pathways include various channel/pump proteins, enzymatic and trafficking processes [see Table 1 in (202)]. These components are widely distributed in the inner ear, including the hair cells, supporting cells, transitory epithelium, dark cells and epithelial cells lining vestibular cavities and the endolymphatic sac. Otoconial crystals have a distinct central core consisting of an organic matrix with a lower level of Ca^2+^, surrounded by largely inorganic peripheral zones with high Ca^2+^ concentrations. Otoconin-90, OC90, is the main component of otoconia (203); OC90 is extremely acidic, favoring the binding of calcium or calcium carbonate (CaCO_3_). This protein also interacts with and selectively recruits other otoconins (e.g., otolin-1, fetuin, osteopontin) to form the initial matrix facilitating the seeding process (204). The continuous addition of organic and inorganic components and the fusion of several minute crystallites ensure correct growth (201, 205). The core, periphery and external surface of the otoconia are interconnected with a fibrous material of various diameters and organizations. One of the major components of these fibers is otolin (also known as otolin-1), an inner-ear secreted glycoprotein that probably forms a collagen-like scaffold for optimal otoconium formation (201, 205).

The otoconial crystals are anchored to the sensory epithelia by the otoconial membrane, a honeycomb-like structure composed of collagenous proteins, such as otolin, and non-collagenous glycoproteins, including otogelin (206, 207), otogelin-like, α-tectorin, β-tectorin (208), and otoancorin (209). Otogelin is required for the anchoring of the otoconial membrane and cupula to the corresponding sensory epithelia. Otogelin-mutant mice have displaced acellular matrices and severe vestibular deficits (210). Mice lacking α-tectorin have few otoconial membranes, with fewer and larger otoconia, but no obvious vestibular behavioral deficits (208). By contrast, no structural or behavioral defects have yet been identified in mice lacking either β-tectorin or otoancorin (208, 211). Mutations of the genes encoding OTOG (otogelin, DFNB18B), OTOGL (otogelin-like, DFNB84B), and TECTA (α-tectorin, DFNA8/12, DFNB21) have been associated with hearing loss in humans, with occasional reports of vestibular hyporeflexia or vertigo in patients with OTOG or TECTA defects.

The development of otoconial biocrystals is regulated by various factors creating an appropriate environment for normal crystal seeding and growth, with correct concentrations of calcium, carbonate, and hydrogen ions in the endolymph above the utricular and saccular maculae. Severe otoconium losses, a complete absence of otoconia and otoconial abnormalities have been reported in mutant mice with an aberrant endolymph ionic composition (i.e., Ca^2+^, H^+^, ${\text{HCO}}_{3}^{-}$, and Na^+^, K^+^, Cl^−^) (201, 202, 205). The list of key players required for the maintenance of crystal structure and the prevention of crystal degeneration include: (i) PMCA2, the plasma membrane Ca^2+^ ATPase 2 (ATP2B2) pump, the main source of Ca^2+^ ions for the formation and maintenance of otoconial crystals; (ii) the Cl^−^/${\text{HCO}}_{3}^{-}$ transporter pendrin, which secretes the bicarbonate required for CaCO_3_ formation and ensures a normal pH by regulating the levels of ${\text{HCO}}_{3}^{-}$ and/or Cl^−^ ions; (iii) the alpha2 delta (α_2_δ_1_)-like ancillary subunit, which modulates Cav3 T-type CACHD1 channels by modulating voltage-gated calcium channels; this subunit contributes to the establishment of appropriate calcium concentrations in regions of otoconia formation (212); (iv) OTOP1, a multiple transmembrane (TM) protein that has been shown to regulate protein secretion and cellular calcium levels in vestibular supporting cells (213); (v) the membrane-bound enzymes NADPH oxidases (Noxs), NADPH oxidase 3 (Nox3), and associated proteins (p22, NOXO1), which produce reactive oxygen species, and contribute to calcium signaling; and (vi) zinc transporter 4 (SLC30A4), which is required for the normal expression of carbonic anhydrase (CA), which helps to maintain appropriate ${\text{HCO}}_{3}^{-}$ concentrations and pH (201, 205).

Additional studies are required to determine the precise mechanisms involving these proteins and to identify other key actors in disorders involving otoconia, otoconial membranes, and/or vestibular epithelia-related disorders. However, more specific, and appropriate clinical and experimental tools are required for thorough, in-depth evaluations of vestibular function, to elucidate the underlying mechanistic features. Naturally arising mutations in more than 340 genes have been reported to cause inner ear malformation or dysfunction in mice [Table S2 in (96)], but these genes only partially overlap with the more than 200 genes identified in humans. Ongoing programs, such as the International Mouse Phenotyping Consortium (IMPC), aiming to produce null alleles by deleting an early critical exon in each mouse gene (214, 215), will significantly increase the number of mutant mice with hearing and balance/vestibular deficits available (216–218). By early 2021, 9,719 knockout mice had been generated, 7,455 of which had been phenotyped through the IMPC comprehensive phenotyping pipeline including inner ear measurements (214), These mice are available to investigators worldwide (http://www.mousephenotype.org/) (215). More thorough studies of these models, with dedicated experimental tools for vestibular evaluations, should improve our understanding of the properties and singularities of vestibular organs.

## Rehabilitation and Treatment Strategies for Vestibular Disorders

Despite this high degree of medical need, as it was discussed in the introduction, the management of patients with vertigo and dizziness still suffers from a lack of diagnostic tools to guide therapeutic management and a lack of effective treatments. Current treatments for vestibular diseases are restricted to pharmacological treatments, rehabilitation, and surgery protocols. All aim to alleviate acute vestibular symptoms, enhance compensation, protect against symptoms aggravation, and promote vestibular rehabilitation. Innovative solutions with promising applications, such as vestibular implants and gene therapy approaches, are being tested in clinical trials.

### Pharmacological Treatments

Pharmacological approaches are frequently considered in the management of vestibular disorders. However, for most, the molecular basis of action remains unknown, efficacy is insufficient or even absent, and many anti-vertigo treatments have significant side effects. In the vestibular end organs, the drugs acting on the vestibular apparatus have diverse cellular targets, including the homeostasis of liquids and electrolytes in the inner ear, the regulation of blood flow, cell homeostasis and survival, and sensory processes related to vestibular information flow. In the vestibular nuclei, drugs act on homeostasis and cell survival, neurotransmitter receptors and ion channel modulation (219). For an in-depth review on the mechanism of action of the drugs used to treat vestibular disorders (219, 220).

### Rehabilitation Treatment

Damage to the vestibular endorgans induces deafferentation of vestibular nucleus leading to a strong disorganization of the vestibular system. In the acute stage, such disturbances induce both static and dynamic vestibular deficits, causing postural, perceptual and oculomotor syndromes. Following vestibular damage, a spontaneous process, referred to as central compensation, allows substantial restoration of posture and balance in patients. It refers to all process by which the brain adapts to changes in inner ear vestibular organs function. The effectiveness of this process is highly case-dependent and its precise cellular and molecular mechanisms remain partially understood so far (221–224). What we know is that the central compensation mechanisms lead to the reorganization of the neuronal pathways and to the restoration of the activity in the ipsilateral and contralateral vestibular nuclei. Electrophysiological and behavioral investigation in animal models suggest that the compensation of the statis ocular motor and postural deficit results on the fast restoration of a balanced spontaneous resting activity in vestibular nuclei on both sides (through inhibition and facilitation mechanisms). The dynamic symptoms however require that the brain developpes new operating modes through different processes: synaptic remodeling, sensory substitution, or behavioral substitution. Dynamic symptoms improve more slowly and may never fully compensate. Vestibular rehabilitation is a physiotherapy program that accelerate compensation phenomena that are effective but slow and helps to correct inappropriate balance strategies. It is designed to promote vestibular compensation through adaptation, substitution, and habituation (225–227). Rehabilitation is often proposed in cases of acute unilateral vestibular deficits, inappropriate spontaneous compensation, or bilateral vestibular loss.

### Surgery and Vestibular Implants

For patients with persistent defined vertigo symptoms after the exhaustion of pharmaceutical and other conservative treatment options suffering a severely impaired quality of life, surgical procedures are often the only remaining alternative. Function-preserving and destructive surgical procedures may be performed, with or without hearing preservation. However, there is heated debate among ENT specialists as to whether such surgery should be performed. All the available surgical procedures and their possible indications have been summarized in (228).

Apart from these traditional surgical procedures, approaches using electrical stimulation of the vestibule for restoring vestibular function have been developed. Currently, two technically different approaches for vestibular implants have been developed: vestibular co-stimulation with a cochlear implant (combined cochleo vestibular implant) or a stand-alone implant designed for intralabyrinthine stimulation in patients with bilateral vestibular loss but without profound sensorineural hearing loss.

The first vestibular implantation in humans was performed by the Maastricht-Geneva group in 2007, with a device derived from a cochlear implant and the placing of a single vestibular electrode on the posterior ampullary nerve (229). A new cochlear-vestibular implant prototype was developed a few years later ^3^, with a vestibular multichannel electrode associated with a special interface coupling a motion sensor for capturing the signals from a three-axis gyroscope (LYPR540AH; ST Microelectronics; Geneva, Switzerland) with the modulation of baseline electrical activity. Using this prototype, they showed that the vestibular implant could restore the VOR both at mid- (230) and high-frequency angular acceleration (231) (rotation test and head impulse test). They also showed that the implant could effectively suppress oscillopsia, one of the most frequent complaints in patients (232).

Another group from Johns Hopkins has worked on a promising standalone multichannel vestibular implant not combined with a cochlear implant. This device consists of a three-axis motion sensor, worn on the head, to record angular head velocity. It transmits the information, in the form of a modulation of the pulse rate and amplitude of stimulation, through the electrodes implanted in each semicircular canal (233). This group was the first to propose a method for vestibular stimulation 24-h-per-day, 7 days per week, preventing episodes of vertigo when the device is turned ON or OFF and allowing for use of the device outside the laboratory setting. Longitudinal follow-up at 6 months and 1 year after the unilateral implantation of a vestibular prosthesis for bilateral vestibular hypofunction revealed improvements over baseline for measurements relating to posture, gait, and quality of life, but hearing loss in the ear with the implant in all but one participant (234).

In parallel, Rubinstein et al. investigated a fundamentally different category of patients, using vestibular implants to treat patients with Meniere's disease. They designed their device to function as a “vestibular pacemaker” to treat Meniere's disease attacks (235). Unfortunately, although the vestibular stimulator successfully encoded vestibular information in Meniere's patients, it also led to a loss of hearing and vestibular function in the ear with the implant, which is a frequent side effect of vestibular implantation regardless of the technique and device used (236). In a more recent paper, they discussed results of their second-generation vestibular implant and they suggested that the etiology of vestibular loss could have a profound impact on sensitivity of vestibular afferents as opposed to cochlear afferents and that this could impact the feasibility of effective vestibular prosthetic devices (237).

In addition to these prototypes, designed to stimulate the semicircular canals, other efforts have been made to develop an implant for direct otolith stimulation (238).

### The Promise of Gene Therapy for Treating Vestibular Deficits

The abovementioned rehabilitation treatments help to alleviate balance deficits and related symptoms, but curative treatments for vestibular disorders are lacking. The momentum gained in gene therapies over the last decade has spurred on the development of many applications in sensory organs. The inner ear, with its easy access, self-contained compartments, and immune-privileged status, is suitable for multiscale functional and behavioral evaluations of disease progression and beneficial outcomes of treatment. The first applications of gene therapy focused on gene replacement (or gene supplementation) therapy, in which a healthy copy of a defective gene is introduced into cells bearing the mutation. These studies essentially targeted key deafness genes small enough to be delivered with adeno-associated virus (AAV) vectors (maximum capacity of 4.7 kb for a single AAV) (49, 89, 239–241) (see Table 1). In recent years, new generations of AAVs with higher transduction rates for inner ear hair cells have been tested (139, 141, 146, 240, 242). Below, we detail some key attempts at cell-specific delivery for hearing and vestibular disorders.

Several recent studies have published promising results for the use of gene therapy to restore auditory and vestibular function in mouse models of Usher syndrome (159). Three of the mutant mice modeling Usher syndrome and displaying significant vestibular dysfunction have defects of genes small enough to fit into a single AAV: harmonin, sans, and whirlin (159). In *Ush1c* knock-in mutant mice, Pan et al. showed that a single injection of *AAV2/Anc80L65-CMV-Ush1c* via the round window membrane in neonatal animals (P0–P1) resulted in a mean rescue of auditory thresholds to within 20 dB of wild-type values for low-to mid-frequency hearing, whereas high-frequency hearing thresholds remained high (139). In a similar approach, a single injection of *AAV8-Ush1g* in *Ush1g* mutant mice totally restored vestibular function, as demonstrated by the restoration of VOR responses and a decrease in circling behavior (144). Focusing on whirlin, defects of which cause isolated hearing loss, and USH2D, Isgrig et al. showed that AAV8-CMV-*Whrn*, delivered via the posterior semicircular canal, successfully restored the morphology of stereociliary bundles and increased hair cell survival in both the treated cochlea and utricle of *Whrn*^*wi*/*wi*^ mice (146). AAV-mediated whirlin expression was found to increase the length of the stereocilia in utricular hair cells and in the cochlear inner hair cells, and to promote hair cell survival in treated *Whrn*^*wi*^ mice (146). Hearing recovery was only partial, with ABR thresholds at 60–70 dB SPL in some treated mutant mice, but vestibular function improved significantly, as demonstrated by a decrease in circling behavior and improvements in the performance of the animal in rotarod and swim tests. The posterior semicircular canal delivery approach resulted in better auditory and vestibular recovery than delivery via the cochlear round window, probably due to a higher hair-cell transduction efficiency following injection into the endolymphatic space (146). Other AAVs have been tested for improving viral transduction in the inner ear. They include exosome-associated AAVs (exo-AAV1, and exo-AAV9), which were used in a mutant mouse defective for *LHFPL5*, a key component of the MET machinery (141). Following the injection of exo-AAV1-CBA-*Lhfpl5* into the inner ears of P1–P2 *Lhfpl5* knockout mice, partial hearing recovery was observed (improvements of up to 30 dB SPL at frequencies of 4–22 kHz). Vestibular function also improved in the *Lhfpl5* knockout mice, as shown by a decrease in head tossing and circling behaviors (141).

However, the limited packaging capacity of AAVs (<4.7 kb for a single AAV) limits the expansion of this delivery approach to many deafness-balance causal genes. Alternative approaches involving dual AAVs, or RNA-based therapies have also been explored to overcome this AAV limitation (49, 240, 243). Splice-switching antisense oligonucleotides (ASO) have been successfully used to target the USH1C messenger RNA (mRNA) transcribed in mice homozygous for the *Ush1c c.216G* > *A* mutation (135, 137). Recent studies include direct assessments of vestibular function based on vestibular sensory evoked potentials (VsEPs). VsEP responses are totally absent in untreated mice and were restored to near-normal levels after injection of the oligonucleotide into neonatal mice. More detailed explorations have shown that ASO-29 delivery to the inner ear improves cochlear hair-cell transduction currents, restores vestibular afferent irregularity, spontaneous firing rate, and sensitivity to head rotation, and successfully restores hearing thresholds and balance-related behaviors in USH1C mice (135, 137). Treatment at P5 and P15 was minimally effective at rescuing vestibular function, but vestibular recovery was nevertheless sufficient to support normal balance-related behaviors, suggesting positive therapeutic effects on balance (135, 137). Additional studies are underway to determine the best delivery approach to the inner ear, the optimal therapeutic window that enables the best beneficial outcomes, and stability of hearing and/or balance recovery overtime.

The recent advent of gene-editing techniques has paved the way for the development of gene therapy for rare diseases. Gene editing provides opportunities to obtain long-term therapeutic benefits regardless of the size of the gene or the nature of the mutation. This new strategy has already been tested and has yielded promising auditory outcomes for approaches targeting the *TMC* gene encoding the MET channel. AAV(Anc80L65)-mediated delivery of the SaCas9-KKH-gRNA complex efficiently prevented deafness in *Bth* mice for up to 1-year post injection (244). DPOAE thresholds in injected Tmc1Bth mice revealed the preservation of OHC function at lower frequencies (5–11 kHz) at 12 weeks of age, and up to 24 weeks of age in the surviving animals. In treated mice, the hair bundles of cochlear OHCs and IHCs (in the 8 and 16 kHz regions) and VHCs recovered a normal morphology after treatment, with minimal hair-cell loss. With steady progress and the improvement of gene-editing tools (new generations of CRISPR/Cas nucleases, base and prime editors) (49, 243, 245, 246), we anticipate increasing interest in the study of balance deficits in mouse models of vestibular dysfunction.

### The Inner Ear Organoids for 3D-Modeling of Human Disorders and Therapeutic Applications

Attempts have been made to reactivate hair-cell regeneration in the inner ear, through Atoh1 overexpression (31), or the transplantation of human induced pluripotent stem cell (hiPSC) cells (247). The recent progress in hiPSC derived organoids make it possible to obtain 3D-models of human organ development and study the disease in human relevant cellular and genomic contexts (248–251). Despite some limitations to obtain mature cochlear tissues, current protocols can be used to obtain type I or type II hair cells in 3D-inner ear organoids (249, 251, 252). Further studies are needed to identify key pathways for optimal control of cell differentiation into supporting cells, neurons and/or hair cells according to downstream applications. Scalable hiPSC-based platforms can be designed to ensure a reliable source of cells for transplantation and cell therapy. Also, by modeling specific hearing and vestibular disorders, inner ear organoids can allow the establishment of clear molecular and cellular phenotypic features, which can be used as read-outs to test potential beneficial outcomes of regenerative or neuroprotective drugs to hearing and/or balance deficits. In this quest of curative treatments for hearing and/or balance disorders, combined information based on animal and human cellular models will likely help improve the potential beneficial outcomes of treatment in preclinical models, prior to clinical trials.

## Conclusion

Clinical surveys worldwide clearly show that vestibular dysfunction is more prevalent than previously thought. This growing awareness of the importance of the vestibular system is timely and is driving efforts to dissect and understand the multiple contributions of the vestibular system. With current clinical tests for balance deficits, it is easy to perform a complete vestibular assessment in clinical practice, even in infants. Vestibular testing should always be proposed if hearing loss is discovered. However, current clinical tests can detect only gross vestibular behavioral defects, and it remains challenging to detect subtle changes in vestibular function and to identify the precise source of the vestibular deficits experienced by patients, in the otolithic organs, cristae, or beyond, in specific neuron subsets of the central vestibular system. More sensitive clinical tests and better software programs for data interpretation are required for accurate determinations of the extent and severity of the balance deficits detected. Various machine learning algorithms are now being implemented for image-based analysis and clinical diagnosis, to predict the presence of peripheral vestibular dysfunction from posturography parameters (253, 254). In this context, the development of new artificial intelligence (AI) tools will be required, to disentangle the intricate contributions of the vestibular, visual, and somatosensory systems, to facilitate the processing and integration of inputs and parameters from multiple sensory modalities (255). These tools may also have applications extending to the identification of clinical endophenotypes, facilitating the precise and accurate definition and classification of vestibular disorders, improving disease profiling and the prediction of disease progression, and guiding decisions about the type or utility of potential treatments.

Animal models presenting specific balance defects will continue to be instrumental in attempts to refine existing tests or to develop new exploratory paradigms adapting tests to the source of the deficit. In addition to the existing mouse models corresponding to most of the human deafness causal genes identified to date (49, 50), the development of many new models is anticipated, through ongoing international collaborative programs [see (132, 217); http://www.mousephenotype.org/]. These animal models are available to researchers worldwide. They allow a full evaluation of the function of all five vestibular endorgans, through the use of a battery of different methods, guaranteeing a high quality of vestibular testing, combined with morphomolecular investigations to ensure the correct interpretation of the scientific results. These models should improve our understanding of the properties and singular features of vestibular disorders, and provide further insight into the evolution, development, physiology and behavior of the vestibular system. These discoveries will also help guide patient management and the search for possible treatments, through studies of the time window for effective treatment, possible injection routes, and long-term treatment stability.

Gene therapy tools have progressed considerably [improved AAVs, new delivery strategies, emerging gene editing tools; see (49, 89, 239–241)] and there is an urgent need for their clinical application to hearing and balance disorders. Consistent with the considerable phylogenetic gap between vestibular and cochlear organs, the intermediate step toward successful gene therapies for the inner ear is now being achieved, at least in preclinical animal models presenting balance deficits. Thanks to recent progress in viral design, most currently available AAVs can mediate high and robust transduction rates of the VHCs in all vestibular compartments (139, 144, 146, 240, 256, 257), ensuring the efficient delivery of therapeutic agents to vestibular target cells. As described above, in almost all gene therapy studies carried out in deaf mouse mutants displaying vestibular deficits, normal balance function was successfully achieved, whereas outcomes for hearing were much more variable (49). Efforts are being made to improve the existing vectors and to identify new vectors providing better control and higher rates of auditory hair cell transduction, especially for the outer hair cells (highly specialized cells unique to mammals). Restoration of the unique properties of the OHCs, such as somatic electromotility or the well-organized shape and staircase pattern of stereocilia, and high-frequency hearing, remains challenging. In the quest for 3D organoids for inner ear organs, it remains difficult to obtain cochlear tissues, but major progress has been made toward reproducing the ontogeny of balance organs and production of vestibular-like hair cells. For these reasons, the vestibular system provides more opportunities for disentangling the signaling pathways for mammalian hair-cell regeneration, setting the stage for the implementation of treatment solutions for restoring normal balance. Gene therapies for balance deficits are now accessible and we expect an increasing number of successful proof of concept studies in animal models with balance deficits. Such studies can include research for possible combined tissue and engineering therapies, coupling the use of gene therapy with current prosthetic devices (hearing aids, cochlear and vestibular implants). Today's efforts to implement and improve these therapeutic interventions in animal models will better prepare their possible transfer into clinics and/or help implement rehabilitation and physical therapies for alleviating hearing and balance symptoms. The path is clear, but success can only be achieved through coordinated actions by scientists across many fields (AI, physics, virology, chemistry, immunology, molecular and cell biology, and physiology), along with joint efforts with AI researchers, engineers, audiologists, clinicians and all actors in the hearing and balance fields.

## Author Contributions

AM and AE-A wrote the first draft of the manuscript. AM, SV, and AE-A reviewed and critically revised the manuscript. All authors contributed to the article and approved the submitted version.

## Funding

The work in the authors' laboratories was funded by the French National Research Agency (ANR), as part of the Second Investissements d'Avenir Program (light4deaf, ANR-15-RHUS-0001), and LabEx LIFESENSES (ANR-10-LABX-65), Fondation pour l'Audition (FPA-19-Stg), ANR-HearInNoise-(ANR-17-CE16-0017), LHW-Stiftung, Fondation de France, Fondation Maladies Rares, & Retina-France. We acknowledge the support of the Institut de l‘Audition by Fondation pour l'Audition.

## Conflict of Interest

The authors declare that the research was conducted in the absence of any commercial or financial relationships that could be construed as a potential conflict of interest.

## Publisher's Note

All claims expressed in this article are solely those of the authors and do not necessarily represent those of their affiliated organizations, or those of the publisher, the editors and the reviewers. Any product that may be evaluated in this article, or claim that may be made by its manufacturer, is not guaranteed or endorsed by the publisher.

## Footnotes

^1^Available online at: https://balanceanddizziness.org/disorders/vestibular-disorders/vestibular-toxicity/.

^2^Available online at: https://dizziness-and-balance.com/disorders/bilat/ototoxins.html.

^3^Available online at: https://patentscope.wipo.int/search/en/detail.jsf?docId=WO2014118094.
