# Supplementary material for: Vestibular Deficits in Deafness: Clinical Presentation, Animal Modeling, and Treatment Solutions
Source: Front Neurol. 2022 Apr 4;13:816534. doi: 10.3389/fneur.2022.816534 (PMC9013928; doi:10.3389/fneur.2022.816534)
Supplement: Supplementary file 1 [file Table_1.pdf]

Human non syndromic and syndromic hearing loss with vestibular dysfunction

Human related SNHL (OMIM)	Gene symbol	Location	Ear pathology/ gene function	Vestibular findings in human	Vestibular findings in animal models	References
**NON SYNDROMIC HEARING LOSS**						
DFNA1 (602121)	*DIAPH1*	5q31.3	Hair bundle development and functioning	Vestibular dysfunction not fully penetrant. Findings consistent with endolymphatic hydrops.		[1]
DFNA3 (121011-604418)	*GJB2 – GJB6*	13q12.11	Cochlea ion homeostasis	Vestibular dysfunction not fully penetrant. Otolithic and canalar dysfunction.	Reduced inner ear size, abnormal distribution of otoliths and reduced otolith size and developmentally delayed semicircular canals in cx30.3 zebrafish.	[2-4]
DFNA6/14 (606201)	*WFS1*	4p16.1	Oxidative stress, metabolims & mitochondria	Vestibular dysfunction in few patients.	Vestibular dysfunction in the mouse model (*tilted – tlt*): complete lack of otoconia in utricule and saccule.	[5, 6]
DFNA7 (600298)	*LMX1A*	1q23.3	Hair cell's adhesion and maintenance	About half of the affected individuals display vestibular dysfunction.	Vestibular dysfunction in the mouse model (*Lmx1a*): circling behavior, impaired righting reflex, hyperactivity.	[7-9]
DFNA9 (603196)	*COCH*	14q12	Transmembrane or secreted proteins and extracellular matrix	Variable vestibular dysfunction up to severe vestibular deficit/areflexia.	Vestibular dysfunction in the mouse model: abnormal vestibular evoked potentials	[10-18]
DFNA11 (276903)	*MYO7A*	11q13.5	Hair bundle development and functioning	Vestibular dysfunction not fully penetrant. Variable degree of vestibular dysfunction.		[19-21]
DFNA13 (120290)	*COL11A2*	6p21.32	Transmembrane or secreted proteins and extracellular matrix	Various caloric abnormalities observed in about half of the subject		[22]
DFNA15 (602460)	*POU4F3*	5q32	Hair cell's adhesion and maintenance	Vestibular dysfunction not fully penetrant. Mild to severe vestibular dysfunction.	*Pou4f3*$^{ddl/ddl}$ mice exhibit vertical head tossing, circling and general hyperactive behavior indicative of a vestibular dysfunction	[23-25]
DFNA17 (160775)	*MYH9*	22q12.3	Hair cell's adhesion and maintenance	Associated to cochleosaccular degeneration.		[26]
DFNA20/26 (102560)	*ACTG1*	17q25.3	Hair bundle development and functioning	Vestibular dysfunction not fully penetrant.		[27]

				Mild to severe hyporeflexia.		
DFNA22 (600970)	*MYO6*	6q14.1	Hair bundle development and functioning		Vestibular dysfunction in mouse with MYO6 mutation : the *Snell's waltzer* (sv) mouse and *Turner* (Tur) mouse.	[28, 29]
DFNA28 (608576)	*GRHL2*	8q22.3	Transcriptional regulation		Vestibular malformation and severe vestibular dysfunction in zebrafish model.	[30]
DFNA41 (600844)	*P2RX2*	12q24.33	Cochlea ion homeostasis		Vestibular dysfunction observed in knock-in mouse model of DFNA41: impaired balance on rotarod and beam walking.	[31]
DFNA69 (184745)	*KITLG*	12q21.32	Transmembrane or secreted proteins and extracellular matrix	Vestibular dysfunction not fully penetrant.		[32]
DFNA76 (602734)	*PLS1*	3q23	Hair bundle development and functioning		Thresholds for vestibular evoked potentials are significantly elevated in mice lacking functional PLS1.	[33]
DFNA78 (610981)	*SLC12A2*	5q23.3	Cochlea ion homeostasis	Vestibular dysfunction with delayed motor skills.	Severe vestibular dysfunction observed in *Slc12a2*$^{-/-}$ mice: head bobbing, circling, collapse of vestibular compartments and epithelia.	[34-36]
DFNB1 (121011 – 604418)	*GJB2* -GJB6 (Cx26 - Cx30)	13q12.11	Cochlea ion homeostasis	Vertigo reported by approximately half of the affected subjects.	Reduced inner ear size, abnormal distribution of otoliths and reduced otolith size and developmentally delayed semicircular canals in cx30.3 zebrafish.	[4, 37]
DFNB2 (276903)	*MYO7A*	11q13.5	Hair bundle development and functioning	Vestibular dysfunction not fully penetrant.		[38]
DFNB3 (602666)	*MYO15A*	17p11.2	Hair bundle development and functioning		Vestibular dysfunction in mouse model of DFNB3 *shaker-2*: head-tossing, circling behavior	[39, 40]
DFNB4 (605646)	*SLC26A4*	7q22.3	Cochlea ion homeostasis	Enlarge vestibular aqueduct and possible Mondini dysplasia. Vestibular dysfunction not fully penetrant.		[41]

DFNB6 (607237)	*TMIE*	3p21.31	Hair bundle development and functioning		Vestibular dysfunction in mouse model of DFNB6 *spinner*: head-shaking, circling behavior.	[42]
DFNB8/10 (605511)	*TMPRSS3*	21q22.3	Cochlea ion homeostasis	Vestibular dysfunction not fully penetrant. Mild vestibular dysfunction.	*Tmprss3*^{Y260X} mice display mild vestibular syndrome that correlated histologically with a slow degeneration of saccular hair cells.	[43, 44]
DFNB16 (606440)	*STRC*	15q15.3	Hair bundle development and functioning	Episodic vertigo. Primarly saccular and utricular dysfunction.		[45]
DFNB18B (604487)	OTOG	11p15.1	Transmembrane or secreted proteins and extracellular matrix	Vestibular dysfunction not fully penetrant. Some individuals have delayed age of walking.	Severe vestibular dysfunction in mouse model with Otog mutation (*Twister*).	[46-48]
DFNB23 (605514)	*PCDH15*	10q21.1	Hair bundle development and functioning		Vestibular dysfunction in animal model of recessive mutations of PCGH15 (*Ames Waltzer* (*av*) mouse)	[49]
DFNB25 (613283)	*GRXCR1*	4p13	Hair bundle development and functioning	Vestibular dysfunction not fully penetrant.		[50]
DFNB31 (607928)	*WRHN*	9q32	Hair bundle development and functioning		Vestibular dysfunction in mouse model of DFNB31 *Whirler (wi)*: circling and head-bobbing.	[51]
DFNB36 (606351)	*ESPN*	1p36.31	Hair bundle development and functioning	Vestibular areflexia.		[52]
DFNB37 (600970)	*MYO6*	6q14.1	Hair bundle development and functioning	Vestibular dysfunction in many individuals with delayed age of walking.		[53]
DFNB59 (610219)	*PJVK*	2q31.2	Oxidative stress, metabolims & mitochondria	Central vestibular dysfunction.		[54]
DFNB68 (605111)	*S1PR2*	19p13.2	Cochlea ion homeostasis		Vestibular dysfunction observed in *S1pr2*$^{-/-}$ mouse: head tilting, poor swimming ability, progressive deterioration of vestibular epithelium and loss or deformity of utricular and saccular otoconia. Vestibular dysfunction in zebrafish model: Structural	[55-59]

					defects are identified within the otic vesicle, the semicircular canals, otoliths, utricle, and saccule.	
DFNB82 (609245)	*GPSM2*	1p13.3	Hair bundle development and functioning		Vestibular dysfunction in mouse model with GPSM2 mutation: hyperactive behavior, circling, short stereocilia.	[60]
DFNB84 (603317)	*PTPRQ*	12q21.31	Hair bundle development and functioning	Severe vestibular dysfunction in all individuals.	Hair bundle defects are observed in the vestibule of *Ptprq*$^{-/-}$ mice.<br><br>Vestibular dysfunction observed in Doberman Pinschers with PTPRQ mutation: head tilt.	[61-63]
DFNB101 (615762)	*GRXCR2*	5q32	Hair bundle development and functioning		Mouse *Grxcr2* deletion mutants exhibit abnormal vestibular evoked potentials.	[64]
DFNB103 (607293)	*CLIC5*	6p21.1	Hair bundle development and functioning<br>Cochlea ion homeostasis	Vestibular dysfunction later in life.	Vestibular dysfunction in mouse model of DFNB103 *Jitterbug (jbg/jbg)*.	[65, 66]
DFNB108 (602336)	ROR1	1p31.3	Synaptic transmission	Fusion of the cochlea and vestibule into a common cavity.		[67]
DFNB109 (612959)	*ESRP1*	1p13.3	Hair cell's adhesion and maintenance	Vestibular dysplasia.	Mouse Esrp1$^{-/-}$: common crus and lateral semicircular canal dysgenesis.	[68]
DFNB110 (603196)	*COCH*	14q12	Transmembrane or secreted proteins and extracellular matrix	Two individuals tested in their first decade of life: one with mild vestibular impairment, the other normal vestibular function.		[69]
DFNB111 (604873)	*MPZL2*	11q23.3	Hair cell's adhesion and maintenance	Slight modifications of the vestibular function.		[70]
DFNX2 (300039)	*POU3F4*	Xq21.1	Transcriptional regulation	Middle and inner ear malformation with perilymphatic Gusher.<br>Severe vestibular dysfunction.	*Pou3f4*-deficient mice: impaired balance, loss of vestibular hair cells	[71, 72]
**SYNDROMIC HEARING LOSS**						
Jervell and Lange-Nielsen syndrome 1	*KCNQ1*		Cochlea ion homeostasis	Severe vestibular dysfunction and delayed motor skills.	Vestibular dysfunction in *Kcnq1*$^{-/-}$ mice: head-bobbing, circling, trouble righting themselves collapse of endolymphatic space.	[73, 74]
Jervell and Lange-Nielsen syndrome 2	*KCNE1*		Cochlea ion homeostasis	Severe vestibular dysfunction and delayed motor skills.	Vestibular dysfunction in *Kcne1*$^{-/-}$ mice: head-	[74, 75]

				bobbing, circling, abnormal development of the endolymphatic space, severe degeneration of the hair cells	
Waardenburg Type 1	*PAX3*	Melanocyte development	Vestibular dysfunction can be observed.		[76]
Waardenburg Type 2	*MITF SNAI2*<br>*SOX10*	Melanocyte development<br>Melanocyte deficiency	Vestibular dysfunction can be observed.<br>Dysmorphic vestibular anatomy linked to SOX10 mutation: bilateral agenesis or hypoplasia of the semicircular canals or both, associated with an enlarged vestibule and a cochlear deformity.		[76, 77]
Waardenburg Type 3	*PAX3*	Melanocyte development			[76]
Waardenburg Type4	*EDNRB*<br>*EDN3*<br>*SOX10*	Melanocyte development	Severe vestibular dysfunction are observed.<br>Dysmorphic vestibular anatomy linked to SOX10 mutation: bilateral agenesis or hypoplasia of the semicircular canals or both, associated with an enlarged vestibule and a cochlear deformity.		[76, 77]
USHER1B	*MYO7a*	Hair bundle development and functioning	Severe vestibular dysfunction with delayed motor skills.	Mouse model (*Shaker*): head-tossing, hyperactivity and circling behaviors. Disorganized stereociliary bundles.<br><br>Zebrafish model (*mariner mutant*): circling swimming behavior. Disorganized stereociliary bundles.	[81-84]
USHER1D	*CDH23*	Hair bundle development and functioning	Severe vestibular dysfunction in most individuals with delayed motor skills.<br>Missense mutations result in either a milder form, which overlaps with clinical types USH2 or 3, or nonsyndromic deafness (DFNB12)	Severe vestibular dysfunction in mouse model of USH1D *Waltzer*: hyperactivity, head-tossing, circling behavior.<br><br>Severe vestibular dysfunction in zebrafish model: balance defect, circling swimming behavior.	[84-88]
USHER1F	*PCDH15*	Hair bundle development and functioning	Severe vestibular dysfunction with delayed motor skills.	Vestibular dysfunction described for mouse with	[49, 84, 89, 90]

				mutation of Pcdh15 *Ames waltzer (*av*)* and distorded stereocilia bundles.<br><br>Vestibular defect in zebrafish Pcdh15 mutants.	
USHER1C	*Ush1c*	Hair bundle development and functioning<br>Synaptic transmission	Severe vestibular dysfunction with delayed motor skills.	Severe vestibular dysfunction in mouse model of USH1C *Waltzer*: hyperactivity, head-tossing, circling behavior and disorganized stereocilia bundles.<br><br>Severe vestibular dysfunction in zebrafish model of USH1C: circling swimming behavior, disorganized stereocilia bundles.	[84, 91-93]
USHER1G	*sans*	Hair bundle development and functioning	Severe vestibular dysfunction with delayed motor skills.<br>Atypical USH1 has also been associated with mutations in the *SANS* gene (USH1G).		[84]
USHER Type 2	ADGRV1<br>VLGR1<br>GPR98<br>*USH2A*<br>*WHRN*	Hair bundle development and functioning	Variable vestibular function.	*Whrn*^{wi} mice and *Whrn*^{neo} mice have severe to profound loss of linear vestibular evoked potential responses and shorter stereocilia.	[94, 95]
USHER Type 3	*CLRN1*<br>*HARS*	Hair bundle development and functioning	Variable vestibular function.	Progressive vestibular loss in Clrn1-/- mice.	[96, 97]
Pendred	*SLC26A4*<br>*EPHA2*<br>*EPHB2*		Dysmorphic vestibular anatomy/ large vestibular aqueduct.<br>Mild vestibular dysfunction primarly in saccul.	Mouse model *Slc26a4*$^{\Delta/\Delta}$ has an enlarged vestibular aqueduct and a Mondini-like dysplasia of the cochlea.<br>Mouse model Ephb2: abnormal otoconia, dysplasia of endolymphatic fluid space, absence of endolymphatic duct.	[98-101]
DRTA	ATP6V1B1<br>ATP6V0A4	Absent endocochlear potential	Large vestibular aqueduct.<br>Vestibular dysfunction can be observed.		[102]

BOR	*EYA1*<br>*SIX1*<br>*SIX5*	Inner ear development	Dysmorphic vestibular anatomy/ large vestibular aqueduct.	Mouse model: head bobbing, vestibular dysmorphy. Reduced number of hair cells.<br><br>Zebrafish model: imbalanced and circling swimming behavior, small otoliths, absent hair cells in cristae, reduced number of hair cells in maculae	[103-108]
22q11 deletion syndrome			Frequent vestibular dysfunction. Frequent inner ear malformations particularly the lateral semi-circular canal .		[109, 110]
CHARGE	*Chd7*<br>*Sema3e*	Inner ear development	Severe vestibular dysfunction with frequent delayed motor skills. Semi-circular canal hypoplasia or agenesis. Vestibulocochlear nerve hypoplasia.	Severe vestibular dysfunction in mouse model: head shaking and circling behavior. Inner ear malformation.	[111-114]

<u>REFERENCES</u>

1. Lalwani, A.K., et al., *Further characterization of the DFNA1 audiovestibular phenotype.* Arch Otolaryngol Head Neck Surg, 1998. **124**(6): p. 699-702.
2. Todt, I., et al., *Vestibular dysfunction of patients with mutations of Connexin 26.* Neuroreport, 2005. **16**(11): p. 1179-81.
3. Melchionda, S., et al., *Functional characterization of a novel Cx26 (T55N) mutation associated to non-syndromic hearing loss.* Biochem Biophys Res Commun, 2005. **337**(3): p. 799-805.
4. Chang-Chien, J., et al., *The connexin 30.3 of zebrafish homologue of human connexin 26 may play similar role in the inner ear.* Hear Res, 2014. **313**: p. 55-66.
5. Hurle, B., et al., *Physical mapping of the mouse tilted locus identifies an association between human deafness loci DFNA6/14 and vestibular system development.* Genomics, 2001. **77**(3): p. 189-99.
6. Kunst, H., et al., *Autosomal dominant non-syndromal low-frequency sensorineural hearing impairment linked to chromosome 4p16 (DFNA14): statistical analysis of hearing threshold in relation to age and evaluation of vestibulo-ocular functions.* Audiology, 1999. **38**(3): p. 165-73.
7. Wesdorp, M., et al., *Heterozygous missense variants of LMX1A lead to nonsyndromic hearing impairment and vestibular dysfunction.* Hum Genet, 2018. **137**(5): p. 389-400.
8. Bergstrom, D.E., L.H. Gagnon, and E.M. Eicher, *Genetic and physical mapping of the dreher locus on mouse chromosome 1.* Genomics, 1999. **59**(3): p. 291-9.

9. Millonig, J.H., K.J. Millen, and M.E. Hatten, *The mouse Dreher gene Lmx1a controls formation of the roof plate in the vertebrate CNS.* Nature, 2000. **403**(6771): p. 764-9.
10. de Kok, Y.J., et al., *A Pro51Ser mutation in the COCH gene is associated with late onset autosomal dominant progressive sensorineural hearing loss with vestibular defects.* Hum Mol Genet, 1999. **8**(2): p. 361-6.
11. Khetarpal, U., *DFNA9 is a progressive audiovestibular dysfunction with a microfibrillar deposit in the inner ear.* Laryngoscope, 2000. **110**(8): p. 1379-84.
12. Usami, S., et al., *Mutations in the COCH gene are a frequent cause of autosomal dominant progressive cochleo-vestibular dysfunction, but not of Meniere's disease.* Eur J Hum Genet, 2003. **11**(10): p. 744-8.
13. Kemperman, M.H., et al., *Audiometric, vestibular, and genetic aspects of a DFNA9 family with a G88E COCH mutation.* Otol Neurotol, 2005. **26**(5): p. 926-33.
14. Pauw, R.J., et al., *Clinical characteristics of a Dutch DFNA9 family with a novel COCH mutation, G87W.* Audiol Neurootol, 2007. **12**(2): p. 77-84.
15. Pauw, R.J., et al., *Phenotype description of a novel DFNA9/COCH mutation, I109T.* Ann Otol Rhinol Laryngol, 2007. **116**(5): p. 349-57.
16. Street, V.A., et al., *A novel DFNA9 mutation in the vWFA2 domain of COCH alters a conserved cysteine residue and intrachain disulfide bond formation resulting in progressive hearing loss and site-specific vestibular and central oculomotor dysfunction.* Am J Med Genet A, 2005. **139A**(2): p. 86-95.
17. Verdoodt, D., et al., *On the pathophysiology of DFNA9: Effect of pathogenic variants in the COCH gene on inner ear functioning in human and transgenic mice.* Hear Res, 2021. **401**: p. 108162.
18. Jones, S.M., et al., *Hearing and vestibular deficits in the Coch(-/-) null mouse model: comparison to the Coch(G88E/G88E) mouse and to DFNA9 hearing and balance disorder.* Hear Res, 2011. **272**(1-2): p. 42-8.
19. Bolz, H., et al., *Impaired calmodulin binding of myosin-7A causes autosomal dominant hearing loss (DFNA11).* Hum Mutat, 2004. **24**(3): p. 274-5.
20. Bischoff, A.M., et al., *Cochleovestibular and ocular features in a Dutch DFNA11 family.* Otol Neurotol, 2006. **27**(3): p. 323-31.
21. Di Leva, F., et al., *Identification of a novel mutation in the myosin VIIA motor domain in a family with autosomal dominant hearing loss (DFNA11).* Audiol Neurootol, 2006. **11**(3): p. 157-64.
22. Kunst, H., et al., *The phenotype of DFNA13/COL11A2: nonsyndromic autosomal dominant mid-frequency and high-frequency sensorineural hearing impairment.* Am J Otol, 2000. **21**(2): p. 181-7.
23. van Drunen, F.J., et al., *Vestibular impairment in a Dutch DFNA15 family with an L289F mutation in POU4F3.* Audiol Neurootol, 2009. **14**(5): p. 303-7.
24. de Heer, A.M., et al., *Mild and variable audiometric and vestibular features in a third DFNA15 family with a novel mutation in POU4F3.* Ann Otol Rhinol Laryngol, 2009. **118**(4): p. 313-20.
25. Hertzano, R., et al., *Transcription profiling of inner ears from Pou4f3(ddl/ddl) identifies Gfi1 as a target of the Pou4f3 deafness gene.* Hum Mol Genet, 2004. **13**(18): p. 2143-53.
26. Lalwani, A.K., et al., *A new locus for nonsyndromic hereditary hearing impairment, DFNA17, maps to chromosome 22 and represents a gene for cochleosaccular degeneration.* Am J Hum Genet, 1999. **64**(1): p. 318-23.
27. Kemperman, M.H., et al., *A Dutch family with hearing loss linked to the DFNA20/26 locus: longitudinal analysis of hearing impairment.* Arch Otolaryngol Head Neck Surg, 2004. **130**(3): p. 281-8.

28. Wong, E.Y., et al., *A Novel ENU-Induced Mutation in Myo6 Causes Vestibular Dysfunction and Deafness.* PLoS One, 2016. **11**(5): p. e0154984.
29. Melchionda, S., et al., *MYO6, the human homologue of the gene responsible for deafness in Snell's waltzer mice, is mutated in autosomal dominant nonsyndromic hearing loss.* Am J Hum Genet, 2001. **69**(3): p. 635-40.
30. Han, Y., et al., *Grhl2 deficiency impairs otic development and hearing ability in a zebrafish model of the progressive dominant hearing loss DFNA28.* Hum Mol Genet, 2011. **20**(16): p. 3213-26.
31. Chen, X., et al., *Generation and characterization of a P2rx2 V60L mouse model for DFNA41.* Hum Mol Genet, 2021. **30**(11): p. 985-995.
32. Zazo Seco, C., et al., *Allelic Mutations of KITLG, Encoding KIT Ligand, Cause Asymmetric and Unilateral Hearing Loss and Waardenburg Syndrome Type 2.* Am J Hum Genet, 2015. **97**(5): p. 647-60.
33. Krey, J.F., et al., *Plastin 1 widens stereocilia by transforming actin filament packing from hexagonal to liquid.* J Cell Biol, 2016. **215**(4): p. 467-482.
34. Delpire, E., et al., *Deafness and imbalance associated with inactivation of the secretory Na-K-2Cl co-transporter.* Nat Genet, 1999. **22**(2): p. 192-5.
35. Flagella, M., et al., *Mice lacking the basolateral Na-K-2Cl cotransporter have impaired epithelial chloride secretion and are profoundly deaf.* J Biol Chem, 1999. **274**(38): p. 26946-55.
36. McNeill, A., et al., *SLC12A2 variants cause a neurodevelopmental disorder or cochleovestibular defect.* Brain, 2020. **143**(8): p. 2380-2387.
37. Dodson, K.M., et al., *Vestibular dysfunction in DFNB1 deafness.* Am J Med Genet A, 2011. **155A**(5): p. 993-1000.
38. Hildebrand, M.S., et al., *Variable hearing impairment in a DFNB2 family with a novel MYO7A missense mutation.* Clin Genet, 2010. **77**(6): p. 563-71.
39. Liang, Y., et al., *Genetic mapping refines DFNB3 to 17p11.2, suggests multiple alleles of DFNB3, and supports homology to the mouse model shaker-2.* Am J Hum Genet, 1998. **62**(4): p. 904-15.
40. Probst, F.J., et al., *Correction of deafness in shaker-2 mice by an unconventional myosin in a BAC transgene.* Science, 1998. **280**(5368): p. 1444-7.
41. Roesch, S., et al., *Genetic Determinants of Non-Syndromic Enlarged Vestibular Aqueduct: A Review.* Audiol Res, 2021. **11**(3): p. 423-442.
42. Mitchem, K.L., et al., *Mutation of the novel gene Tmie results in sensory cell defects in the inner ear of spinner, a mouse model of human hearing loss DFNB6.* Hum Mol Genet, 2002. **11**(16): p. 1887-98.
43. Weegerink, N.J., et al., *Genotype-phenotype correlation in DFNB8/10 families with TMPRSS3 mutations.* J Assoc Res Otolaryngol, 2011. **12**(6): p. 753-66.
44. Fasquelle, L., et al., *Tmprss3, a transmembrane serine protease deficient in human DFNB8/10 deafness, is critical for cochlear hair cell survival at the onset of hearing.* J Biol Chem, 2011. **286**(19): p. 17383-97.
45. Frykholm, C., et al., *Stereocilin gene variants associated with episodic vertigo: expansion of the DFNB16 phenotype.* Eur J Hum Genet, 2018. **26**(12): p. 1871-1874.
46. Schraders, M., et al., *Mutations of the gene encoding otogelin are a cause of autosomal-recessive nonsyndromic moderate hearing impairment.* Am J Hum Genet, 2012. **91**(5): p. 883-9.
47. Yu, S., et al., *A novel early truncation mutation in OTOG causes prelingual mild hearing loss without vestibular dysfunction.* Eur J Med Genet, 2019. **62**(1): p. 81-84.

48. Simmler, M.C., et al., *Twister mutant mice are defective for otogelin, a component specific to inner ear acellular membranes.* Mamm Genome, 2000. **11**(11): p. 961-966.
49. Alagramam, K.N., et al., *The mouse Ames waltzer hearing-loss mutant is caused by mutation of Pcdh15, a novel protocadherin gene.* Nat Genet, 2001. **27**(1): p. 99-102.
50. Mori, K., et al., *Novel mutation in GRXCR1 at DFNB25 lead to progressive hearing loss and dizziness.* Ann Otol Rhinol Laryngol, 2015. **124 Suppl 1**: p. 129S-34S.
51. Mathur, P.D., et al., *A study of whirlin isoforms in the mouse vestibular system suggests potential vestibular dysfunction in DFNB31-deficient patients.* Hum Mol Genet, 2015. **24**(24): p. 7017-30.
52. Naz, S., et al., *Mutations of ESPN cause autosomal recessive deafness and vestibular dysfunction.* J Med Genet, 2004. **41**(8): p. 591-5.
53. Ahmed, Z.M., et al., *Mutations of MYO6 are associated with recessive deafness, DFNB37.* Am J Hum Genet, 2003. **72**(5): p. 1315-22.
54. Ebermann, I., et al., *Truncating mutation of the DFNB59 gene causes cochlear hearing impairment and central vestibular dysfunction.* Hum Mutat, 2007. **28**(6): p. 571-7.
55. MacLennan, A.J., et al., *The S1P2 sphingosine 1-phosphate receptor is essential for auditory and vestibular function.* Hear Res, 2006. **220**(1-2): p. 38-48.
56. Kono, M., et al., *Deafness and stria vascularis defects in S1P2 receptor-null mice.* J Biol Chem, 2007. **282**(14): p. 10690-6.
57. Hu, Z.Y., et al., *Gene miles-apart is required for formation of otic vesicle and hair cells in zebrafish.* Cell Death Dis, 2013. **4**: p. e900.
58. Herr, D.R., et al., *Sphingosine 1-phosphate (S1P) signaling is required for maintenance of hair cells mainly via activation of S1P2.* J Neurosci, 2007. **27**(6): p. 1474-8.
59. Santos-Cortez, R.L., et al., *Autosomal-Recessive Hearing Impairment Due to Rare Missense Variants within S1PR2.* Am J Hum Genet, 2016. **98**(2): p. 331-8.
60. Mauriac, S.A., et al., *Defective Gpsm2/Gα.* Nat Commun, 2017. **8**: p. 14907.
61. Goodyear, R.J., et al., *Hair bundle defects and loss of function in the vestibular end organs of mice lacking the receptor-like inositol lipid phosphatase PTPRQ.* J Neurosci, 2012. **32**(8): p. 2762-72.
62. Schraders, M., et al., *Mutations in PTPRQ are a cause of autosomal-recessive nonsyndromic hearing impairment DFNB84 and associated with vestibular dysfunction.* Am J Hum Genet, 2010. **86**(4): p. 604-10.
63. Guevar, J., et al., *Deafness and vestibular dysfunction in a Doberman Pinscher puppy associated with a mutation in the PTPRQ gene.* J Vet Intern Med, 2018. **32**(2): p. 665-669.
64. Avenarius, M.R., et al., *Grxcr2 is required for stereocilia morphogenesis in the cochlea.* PLoS One, 2018. **13**(8): p. e0201713.
65. Seco, C.Z., et al., *Progressive hearing loss and vestibular dysfunction caused by a homozygous nonsense mutation in CLIC5.* Eur J Hum Genet, 2015. **23**(2): p. 189-94.
66. Gagnon, L.H., et al., *The chloride intracellular channel protein CLIC5 is expressed at high levels in hair cell stereocilia and is essential for normal inner ear function.* J Neurosci, 2006. **26**(40): p. 10188-98.
67. Diaz-Horta, O., et al., *ROR1 is essential for proper innervation of auditory hair cells and hearing in humans and mice.* Proc Natl Acad Sci U S A, 2016. **113**(21): p. 5993-8.
68. Rohacek, A.M., et al., *ESRP1 Mutations Cause Hearing Loss due to Defects in Alternative Splicing that Disrupt Cochlear Development.* Dev Cell, 2017. **43**(3): p. 318-331.e5.
69. JanssensdeVarebeke, S.P.F., et al., *Bi-allelic inactivating variants in the COCH gene cause autosomal recessive prelingual hearing impairment.* Eur J Hum Genet, 2018. **26**(4): p. 587-591.

70. Wesdorp, M., et al., *MPZL2, Encoding the Epithelial Junctional Protein Myelin Protein Zero-like 2, Is Essential for Hearing in Man and Mouse.* Am J Hum Genet, 2018. **103**(1): p. 74-88.
71. Xiang, M., et al., *Essential role of POU-domain factor Brn-3c in auditory and vestibular hair cell development.* Proc Natl Acad Sci U S A, 1997. **94**(17): p. 9445-50.
72. Cremers, C.W. and P.L. Huygen, *Clinical features of female heterozygotes in the X-linked mixed deafness syndrome (with perilymphatic gusher during stapes surgery).* Int J Pediatr Otorhinolaryngol, 1983. **6**(2): p. 179-85.
73. Casimiro, M.C., et al., *Targeted disruption of the Kcnq1 gene produces a mouse model of Jervell and Lange-Nielsen Syndrome.* Proc Natl Acad Sci U S A, 2001. **98**(5): p. 2526-31.
74. Winbo, A. and A. Rydberg, *Vestibular dysfunction is a clinical feature of the Jervell and Lange-Nielsen Syndrome.* Scand Cardiovasc J, 2015. **49**(1): p. 7-13.
75. Wu, X., et al., *Gene therapy via canalostomy approach preserves auditory and vestibular functions in a mouse model of Jervell and Lange-Nielsen syndrome type 2.* Nat Commun, 2021. **12**(1): p. 697.
76. Black, F.O., et al., *A vestibular phenotype for Waardenburg syndrome?* Otol Neurotol, 2001. **22**(2): p. 188-94.
77. Elmaleh-Bergès, M., et al., *Spectrum of temporal bone abnormalities in patients with Waardenburg syndrome and SOX10 mutations.* AJNR Am J Neuroradiol, 2013. **34**(6): p. 1257-63.
78. Deol, M.S., *The relationship between abnormalities of pigmentation and of the inner ear.* Proc R Soc Lond B Biol Sci, 1970. **175**(39): p. 201-17.
79. Hodgkinson, C.A., et al., *Mutations at the mouse microphthalmia locus are associated with defects in a gene encoding a novel basic-helix-loop-helix-zipper protein.* Cell, 1993. **74**(2): p. 395-404.
80. Tassabehji, M., V.E. Newton, and A.P. Read, *Waardenburg syndrome type 2 caused by mutations in the human microphthalmia (MITF) gene [see comments].* Nat Genet, 1994. **8**(3): p. 251-5.
81. Ernest, S., et al., *Mariner is defective in myosin VIIA: a zebrafish model for human hereditary deafness.* Hum Mol Genet, 2000. **9**(14): p. 2189-96.
82. Gibson, F., et al., *A type VII myosin encoded by the mouse deafness gene shaker-1.* Nature, 1995. **374**(6517): p. 62-4.
83. Weil, D., et al., *Human myosin VIIA responsible for the Usher 1B syndrome: a predicted membrane-associated motor protein expressed in developing sensory epithelia.* Proc Natl Acad Sci U S A, 1996. **93**(8): p. 3232-7.
84. Yan, D. and X.Z. Liu, *Genetics and pathological mechanisms of Usher syndrome.* J Hum Genet, 2010. **55**(6): p. 327-35.
85. Bolz, H., et al., *Mutation of CDH23, encoding a new member of the cadherin gene family, causes Usher syndrome type 1D.* Nat Genet, 2001. **27**(1): p. 108-12.
86. Di Palma, F., et al., *Mutations in Cdh23, encoding a new type of cadherin, cause stereocilia disorganization in waltzer, the mouse model for Usher syndrome type 1D.* Nat Genet, 2001. **27**(1): p. 103-7.
87. Söllner, C., et al., *Mutations in cadherin 23 affect tip links in zebrafish sensory hair cells.* Nature, 2004. **428**(6986): p. 955-9.
88. Pennings, R.J., et al., *Variable clinical features in patients with CDH23 mutations (USH1D-DFNB12).* Otol Neurotol, 2004. **25**(5): p. 699-706.
89. Raphael, Y., et al., *Severe vestibular and auditory impairment in three alleles of Ames waltzer (av) mice.* Hear Res, 2001. **151**(1-2): p. 237-249.
90. Barber Phillips, J., et al., *Zebrafish PCDH15 (USH1F) mutants display visual and audiovestibular defects.* Investigative Ophthalmology & Visual Science, 2021. **62**: p. 2259.
91. Lentz, J., et al., *Ush1c216A knock-in mouse survives Katrina.* Mutat Res, 2007. **616**(1-2): p. 139-44.

92. Phillips, J.B., et al., *Harmonin (Ush1c) is required in zebrafish Müller glial cells for photoreceptor synaptic development and function.* Dis Model Mech, 2011. **4**(6): p. 786-800.
93. Smith, R.J., et al., *Clinical variability and genetic heterogeneity within the Acadian Usher population.* Am J Med Genet, 1992. **43**(6): p. 964-9.
94. Mathur, P.D. and J. Yang, *Usher syndrome and non-syndromic deafness: Functions of different whirlin isoforms in the cochlea, vestibular organs, and retina.* Hear Res, 2019. **375**: p. 14-24.
95. Magliulo, G., et al., *Usher's Syndrome Type II: A Comparative Study of Genetic Mutations and Vestibular System Evaluation.* Otolaryngol Head Neck Surg, 2017. **157**(5): p. 853-860.
96. Joensuu, T., et al., *Mutations in a novel gene with transmembrane domains underlie Usher syndrome type 3.* Am J Hum Genet, 2001. **69**(4): p. 673-84.
97. Geng, R., et al., *Usher syndrome IIIA gene clarin-1 is essential for hair cell function and associated neural activation.* Hum Mol Genet, 2009. **18**(15): p. 2748-60.
98. West, N.C., A.C. Ryberg, and P. Cayé-Thomasen, *Vestibular Function in Pendred Syndrome: Intact High Frequency VOR and Saccular Hypersensitivity.* Otol Neurotol, 2021. **42**(9): p. e1327-e1332.
99. Wangemann, P., *Mouse models for pendrin-associated loss of cochlear and vestibular function.* Cell Physiol Biochem, 2013. **32**(7): p. 157-65.
100. Raft, S., et al., *Ephrin-B2 governs morphogenesis of endolymphatic sac and duct epithelia in the mouse inner ear.* Dev Biol, 2014. **390**(1): p. 51-67.
101. Li, M., et al., *Digenic inheritance of mutations in EPHA2 and SLC26A4 in Pendred syndrome.* Nat Commun, 2020. **11**(1): p. 1343.
102. Berrettini, S., et al., *Distal renal tubular acidosis associated with isolated large vestibular aqueduct and sensorineural hearing loss.* Ann Otol Rhinol Laryngol, 2002. **111**(5 Pt 1): p. 385-91.
103. Ceruti, S., et al., *Temporal bone anomalies in the branchio-oto-renal syndrome: detailed computed tomographic and magnetic resonance imaging findings.* Otol Neurotol, 2002. **23**(2): p. 200-7.
104. Bosman, E.A., et al., *Catweasel mice: a novel role for Six1 in sensory patch development and a model for branchio-oto-renal syndrome.* Dev Biol, 2009. **328**(2): p. 285-96.
105. Kozlowski, D.J., et al., *The zebrafish dog-eared mutation disrupts eya1, a gene required for cell survival and differentiation in the inner ear and lateral line.* Dev Biol, 2005. **277**(1): p. 27-41.
106. Xu, P.X., et al., *Eya1-deficient mice lack ears and kidneys and show abnormal apoptosis of organ primordia.* Nat Genet, 1999. **23**(1): p. 113-7.
107. Zou, D., et al., *Eya1 gene dosage critically affects the development of sensory epithelia in the mammalian inner ear.* Hum Mol Genet, 2008. **17**(21): p. 3340-56.
108. Song, M.H., et al., *Mutational analysis of EYA1, SIX1 and SIX5 genes and strategies for management of hearing loss in patients with BOR/BO syndrome.* PLoS One, 2013. **8**(6): p. e67236.
109. Willaert, A., et al., *Vestibular dysfunction is a manifestation of 22q11.2 deletion syndrome.* Am J Med Genet A, 2019. **179**(3): p. 448-454.
110. Verheij, E., et al., *Anatomic Malformations of the Middle and Inner Ear in 22q11.2 Deletion Syndrome: Case Series and Literature Review.* AJNR Am J Neuroradiol, 2018. **39**(5): p. 928-934.
111. Bosman, E.A., et al., *Multiple mutations in mouse Chd7 provide models for CHARGE syndrome.* Hum Mol Genet, 2005. **14**(22): p. 3463-76.

112. Jongmans, M.C., et al., *Familial CHARGE syndrome and the CHD7 gene: a recurrent missense mutation, intrafamilial recurrence and variability.* Am J Med Genet A, 2008. **146A**(1): p. 43-50.
113. Aramaki, M., et al., *Phenotypic spectrum of CHARGE syndrome with CHD7 mutations.* J Pediatr, 2006. **148**(3): p. 410-4.
114. Martin, D.M., S. Sheldon, and J.L. Gorski, *CHARGE association with choanal atresia and inner ear hypoplasia in a child with a de novo chromosome translocation t(2;7)(p14;q21.11).* Am J Med Genet, 2001. **99**(2): p. 115-9.
